# Shifted microbial network characteristics govern soil N_2_O emission following paddy-to-vegetable land conversion

**DOI:** 10.3389/fmicb.2026.1750894

**Published:** 2026-01-29

**Authors:** Chenglin Li, Ziqun Zhou, Xin Chen, Quan Tang, Qingbi Zhang, Jieshi Tang

**Affiliations:** 1 Environmental Health Effects and Risk Assessment Key Laboratory of Luzhou, School of Public Health, Southwest Medical University, Luzhou, China; 2 State Key Laboratory of Soil and Sustainable Agriculture, Institute of Soil Science, Chinese Academy of Sciences, Nanjing, China; 3 College of Life Sciences, Shihezi University, Shihezi, China; 4 Key Laboratory of Arable Land Quality Monitoring and Evaluation, Ministry of Agriculture and Rural Affairs, Yangzhou University, Yangzhou, China

**Keywords:** bacterial community, fungal community, land conversion, network complexity, nitrous oxide

## Abstract

Land use conversion from flooded paddy fields to upland vegetable systems is becoming increasingly widespread, yet its ecological consequences for soil N_2_O emissions remain poorly understood. Here, we integrated the potential denitrification-derived N_2_O flux measurements, microbial community profiling, and network analyses to elucidate how paddy-to-vegetable land conversion reshapes soil microbial interactions and regulates N_2_O emission dynamics in the Yangtze River Delta region of China. Results showed that N_2_O emissions increased significantly following the conversion, with fluxes reaching approximately 0.43 and 0.0083 nmol N g^−1^ h^−1^ in soils under vegetable cultivation for 4 and 7 years, respectively. In contrast to the trend in N_2_O emissions, bacterial diversity decreased significantly following the conversion, whereas fungal diversity showed no significant change. Co-occurrence network analysis demonstrated a divergent response of bacterial and fungal communities to land use conversion. In vegetable soils, bacterial networks exhibited enhanced connectivity, with average degrees 1.23 and 1.17 times higher than those in paddy soils after 4 and 7 years of conversion, respectively. Conversely, fungal networks showed markedly reduced connectivity, with average degrees declining by 54.67 and 36.70%, respectively. The number of edges, positive connection edges, negative connection edges, the number of vertices, and average degree in the bacterial network were all significantly positively correlated with N_2_O emission rates, whereas fungal network connectivity showed opposite trends. Random forest modeling further identified bacterial network features were the most influential determinant of N_2_O emissions, outperforming traditional soil environmental variables. Altogether, our findings demonstrate that paddy-to-vegetable land conversion alters the architecture, stability, and modularity of soil microbial networks, which may play a pivotal role in enhanced N_2_O emissions. This study emphasizes the necessity of considering microbial network dynamics in greenhouse gas mitigation strategies.

## 1 Introduction

Nitrous oxide (N_2_O) is a powerful greenhouse gas and a significant ozone-depleting compound, exhibiting a global warming potential roughly 273 times greater than that of carbon dioxide (CO_2_) over a century (Li T. T. et al., 2025). Terrestrial soils, particularly agricultural soils, represent the primary source of N_2_O emissions to the atmosphere (Zhu G. B. et al., 2025), with its production primarily driven by microbial-mediated nitrogen transformations (Xiong et al., 2025). Land use changes, whether involving the conversion of natural vegetation to cropland or the transition between different agricultural systems, modify various properties of soils (Babur et al., 2025), which in turn influence nitrogen cycling and N_2_O emissions (Chen et al., 2025). A meta-analysis spanning tropical and subtropical regions revealed that the shift to fertilized agriculture led to the most significant changes in N₂O flux (Van Lent et al., 2015). Additionally, modeling studies indicate that with increasing nitrogen fertilizer use and land use changes, agricultural lands are increasingly contributing to global soil N_2_O emissions (Ma J. Y. et al., 2025). Given the escalating global shifts in land use practices (Wang et al., 2025), understanding the mechanisms underlying soil N_2_O emissions in response to land use changes is crucial for effective agricultural ecosystem management and climate change mitigation.

In many regions of Asia and elsewhere, paddy fields (flooded rice systems) have been increasingly converted to upland vegetable cultivation as a result of economic pressures, labor changes, water-management issues and land use planning (Wei et al., 2022). This transformation shifts the soil environment from a permanently or seasonally flooded anaerobic state to a more aerobic and intensively managed upland farming system. When land use practices are replaced by intensive cultivation and management, nitrogen mineralization is often accelerated, increasing the availability of inorganic nitrogen (Zhu K. et al., 2025). These changes typically enhance microbial substrate availability, thereby increasing the potential for N_2_O emissions (Li C. L. et al., 2022). Furthermore, shifts in hydrological conditions, redox status, root zone processes, fertilization practices, and tillage intensity contribute to the development of distinct soil microbial communities (Li W. H. et al., 2025), which are expected to have profound impacts on N_2_O production and emission potential.

Traditionally, studies of soil N_2_O emissions have emphasized bulk microbial abundance or key functional genes (Muhammad et al., 2022), such as the *nirK*/*nirS* genes encoding nitrite reductase and the *nosZ* gene encoding N_2_O reductase (Li et al., 2024). Research has shown that after land use changes, increases in the abundance of nitrifying and denitrifying microbial genes often lead to elevated soil N_2_O emissions (Zhang et al., 2022). However, emerging evidence underscores the pivotal role of microbial community interactions, such as species co-occurrence, competition, or cooperation can influence functional outcomes (e.g., N_2_O production or consumption) in ways that extend beyond mere gene abundance (Li et al., 2023). Network-analysis approaches (e.g., co-occurrence networks and molecular ecological networks) provide a framework to characterize microbial community complexity, modularity, connectivity and stability (Baker et al., 2024). This methodology has been demonstrated as an effective tool for exploring microbial community interactions and organization (Faust and Raes, 2012). Research has indicated that the topological features of these networks are influenced by changes in environmental conditions and can serve as indicators of alterations in the ecosystem’s potential functionality (Felipe-Lucia et al., 2020). For instance, Cornell et al. (2023) reported that converting temperate grasslands to conventional croplands increases microbial network complexity (e.g., connectance) as a means of compensating for the ecological functional losses resulting from biodiversity decline. Whereas, in ecosystems under high stress and resource scarcity, microorganisms may survive by employing niche differentiation strategies to promote diverse nutrient utilization, resulting in the formation of additional micro-modules (Yang et al., 2025). Regarding N_2_O emissions in nitrogen transformation processes, it has been demonstrated that in freshwater lakes, the emission levels are significantly influenced by the complexity of denitrifying community networks (Su et al., 2024). These findings suggest that microbial network properties (topology, keystone taxa, modularity, and negative/positive links) may serve as mechanistic levers linking soil environmental change to greenhouse-gas fluxes. Yet explicit investigations of how land use conversion (especially paddy-to-vegetable) reconfigures microbial networks and thereby regulates N_2_O emissions are still scarce.

In China’s Yangtze River Delta region, a globally important agricultural and economic hub, traditional flooded rice fields are increasingly being converted into vegetable fields. This region provides an ideal natural laboratory for investigating how paddy-to-vegetable fields conversion affects soil microbial networks and N_2_O emissions. Previous studies in this region have documented elevated N_2_O emissions following paddy conversion (Wei et al., 2022), but the microbial network mechanisms underlying these changes remain poorly quantified. Given the complex interactions among soil microorganisms, identifying how bacterial and fungal networks respond to this transformation is critical for elucidating the microbial ecological drivers of N_2_O dynamics in this region. Specifically, two major knowledge gaps persist: (1) whether microbial diversity and network structural characteristics respond differently to short-term and long-term land use conversion; (2) how significant are network characteristics for N_2_O emissions under this type of land use change. To address these gaps, this study investigates the responses of bacterial and fungal network characteristics in a rice field and vegetable fields converted from rice field for 4 years and 7 years at the Yangtze River Delta and explores their linkages to soil N_2_O emissions. We hypothesized that (1) the conversion of rice fields to vegetable cultivation increases the complexity and stability of microbial networks, but such enhancement diminishes with prolonged conversion; (2) microbial network characteristics have a significant impact on N_2_O emissions during land use change.

## 2 Materials and methods

### 2.1 Soil sampling

Soil samples were collected from a rice field (RF) and vegetable fields converted from paddy soils for 4 and 7 years (VE4 and VE7, respectively), near the Changshu Agricultural Ecological Experimental Station of the Institute of Soil Science, Chinese Academy of Sciences, in Jiangsu, China. The region traditionally follows a wheat-rice rotation system with N fertilization rates of 500 kg ha^−1^. Driven by economic incentives, some rice fields have been converted to vegetable cultivation, with N application rates typically exceeding 800 kg ha^−1^. At the time of sampling, soil samples were collected from the top 20 cm layer by removing the top 2 cm of soil to eliminate plant residue interference. To minimize the short-term influences of fertilization pulses as well as crop-related disturbances on soil nitrogen transformation processes and microbial communities, soil sampling was uniformly conducted within 1 week after crop harvest. This timing was intended to better capture the background state of soil microbial networks and nitrogen transformation processes across different land use types, thereby enhancing comparability among samples. Three independent sampling locations (≥ 10 m apart) were selected for each land use type, with five random sub-samples taken at each location and thoroughly homogenized to generate one composite sample. Resulting in nine samples in total. After collection, samples were promptly transported to the laboratory and stored at 4 °C for incubation experiments. A portion of the samples was air-dried for analysis of soil properties. The basic characteristics of the soils under different treatments were provided by Li C. L. et al. (2022) and are also presented in Supplementary Table S1.

### 2.2 Soil incubation experiment

Soil N_2_O emission rates were measured using the slurry incubation method (Chen et al., 2019). Soil samples stored in a refrigerator were mixed with He-flushed water at a 1:5 ratio and transferred into 12 mL glass vials (Exetainer; Labco, United Kingdom) in an anaerobic chamber. After sealing, the vials were placed on a vertical shaker and pre-incubated at 25 °C for 1 day to restore soil activity and deplete background oxygen. Then, the slurry was incubated at 25 °C for 7 h, after which 200 μL of saturated ZnCl_2_ was added to each sample to terminate the incubation. Following incubation, the samples were centrifuged, and 5 mL of supernatant was transferred from each vial into 18.5 mL vacuum serum bottles using a syringe. Environmental air was then introduced into the bottles, and they were immediately equilibrated at 5 °C for 24 h. Three milliliters of headspace gas were extracted from each vial with a syringe and analyzed for N_2_O concentration using a gas chromatograph (Agilent 7890A, United States). The total N_2_O concentration in the slurry was estimated based on the measured headspace N_2_O concentration and the Bunsen solubility coefficient at 5 °C. The N_2_O emission rate was subsequently derived from the temporal variation in N_2_O concentrations using linear fitting over the incubation period. It is important to note that since all soil samples were measured under anaerobic conditions, N_2_O emissions are considered a result of denitrification. The N_2_O emission rates measured under anaerobic conditions are regarded as the potential for N_2_O emissions. Therefore, the subsequent N_2_O emission rates reflect only the potential denitrification-derived N_2_O production under controlled anaerobic conditions.

### 2.3 Soil microbial community analysis

Soil samples were freeze-dried, and 0.5 g was used for DNA extraction using the Fast DNA SPIN kit (MP Biomedical, Cleveland, OH, United States). Microbial community composition was analyzed through high-throughput sequencing, with amplicon sequencing performed on an Illumina MiSeq platform (Illumina, San Diego, CA, United States). Amplification of the V4-V5 regions of the universal 16S rRNA gene and the ITS1 region of fungal ITS genes was performed using the corresponding primer sets (Zhu et al., 2024). Following data filtering, 260,892 bacterial sequences and 313,902 fungal sequences were obtained. The bioinformatics analysis process and PCR reaction conditions are described in detail in the Supplementary Table S2. Furthermore, we constructed a co-occurrence network using all samples. After excluding low-abundance groups and performing trimmed mean of M-values (TMM) normalization, spearman correlation coefficients were calculated between taxon (at the genus level). Only pairs exhibiting a correlation coefficient above 0.7 and a *p* value below 0.05 were maintained (Fan et al., 2019; Ma X. et al., 2025). The network and its primary modules were visualized using Gephi.^1^ For each module, microbial group relative abundance was derived from the standardized species abundance (z-score). Linear regression was then applied to assess the relationship between taxon abundance within each module and N_2_O emission rates. Taxa exhibiting high *Zi* values were classified as keystone nodes (Liu et al., 2025). The *Zi* values, network robustness, and subnetwork topology across different treatments were calculated using the ggClusterNet package in R (Wen et al., 2022). Microbial network cohesion was quantified using the microeco package in R (Liu et al., 2023).

### 2.4 Statistical analysis

All statistical analyses, except for the comparison of network robustness differences, were conducted using SPSS (version 27.0). Statistical significance between different land use types was tested using one-way analysis of ANOVA, followed by LSD testing. Differences in microbial network robustness between land use types were compared using a two-sided *t*-test, performed in R software. Differences in microbial communities across distinct land use types were examined using the ANOSIM method. The Pearson correlation between N_2_O emission rates and microbial network topology was calculated using the linkET package in R. The habitat niche breadth of taxa was quantified by the niche.width function from the spaa package (Zheng et al., 2025). And key factors influencing N_2_O emissions were analyzed and assessed using the randomForest package in R. Principal component analysis (PCA) was used to construct composite indices for soil microbial diversity, network characteristics, and soil properties (Chen Q. et al., 2021). The first principal component (the dimension with the highest explained variance) was selected for random forest analysis.

## 3 Results

### 3.1 Changes in N_2_O emissions and microbial community composition

**Figure 1:**
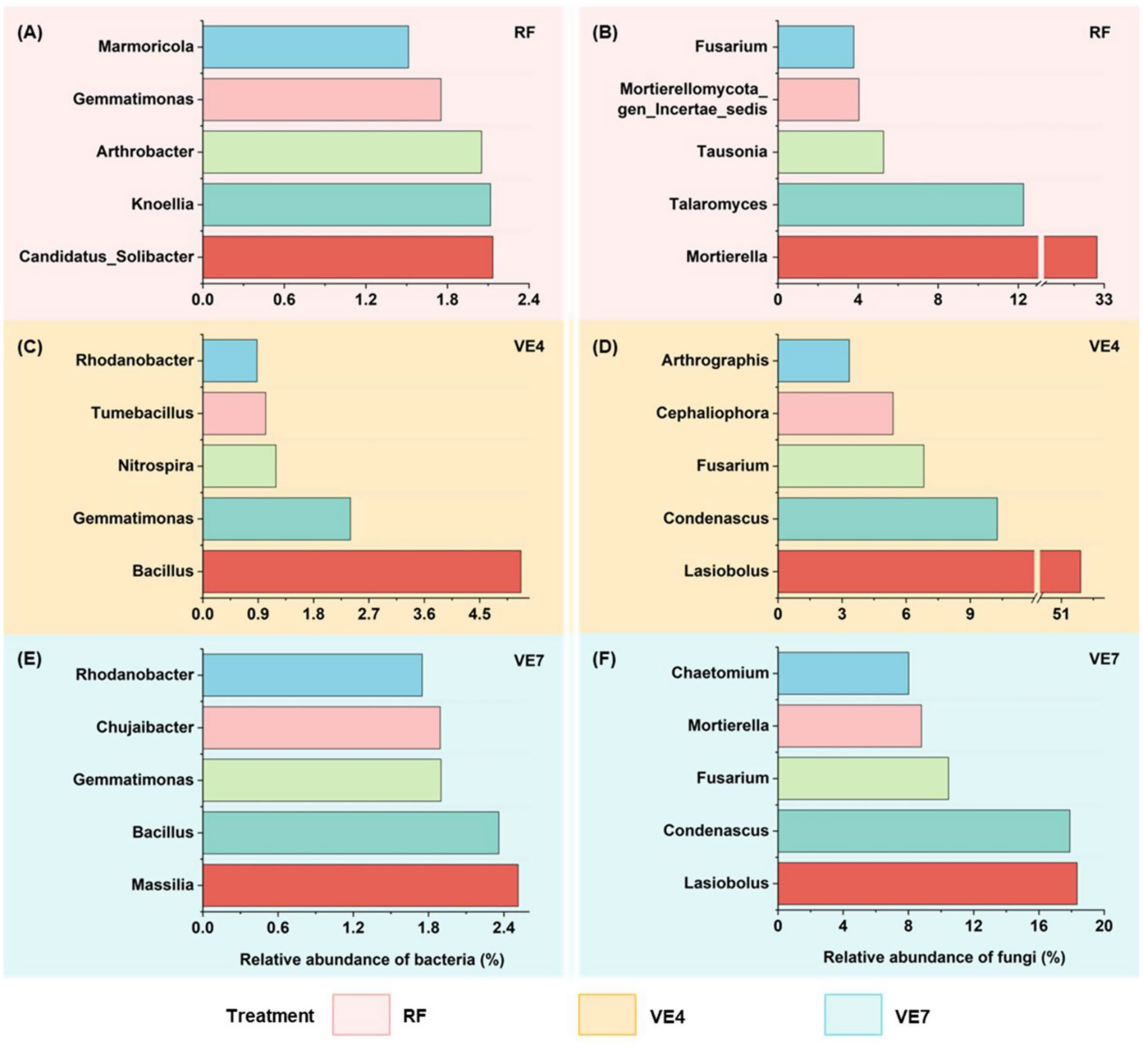
Relative abundances of dominant bacterial **(A,C,E)** and fungal **(B,D,F)** genera in a rice field (RF) and vegetable fields converted from the rice field for 4 years (VE4) and 7 years (VE7).

The conversion of rice fields to vegetable cultivation significantly influenced soil N_2_O emissions (Supplementary Figure S1). Compared to the RF system, both vegetable fields converted from RF for 4 years and 7 years showed elevated N_2_O emission rates. Specifically, the N_2_O emission rates in VE4 were approximately 0.43 nmol N g^−1^ h^−1^ higher than those in the RF system, while the emissions in VE7 were nearly 17.86 times higher. The transition from rice fields to vegetable fields also resulted in shifts in microbial community composition (Figure 1; Supplementary Figure S2). In RF soils, *Candidatus_Solibacter* (2.13%), *Knoellia* (2.12%), and *Arthrobacter* (2.05%) were the most abundant bacterial genera (Figure 1A). In contrast, VE4 and VE7 soils showed a marked increase in the abundance of nitrogen-cycling bacteria (Figures 1C,E), such as *Bacillus* (2.36–5.17%) and *Gemmatimonas* (1.90–2.40%). Additionally, fungal communities also shifted, with an increase in *Lasiobolus*, *Condenascus*, and *Fusarium* (Figures 1B,D,F).

**Figure 2:**
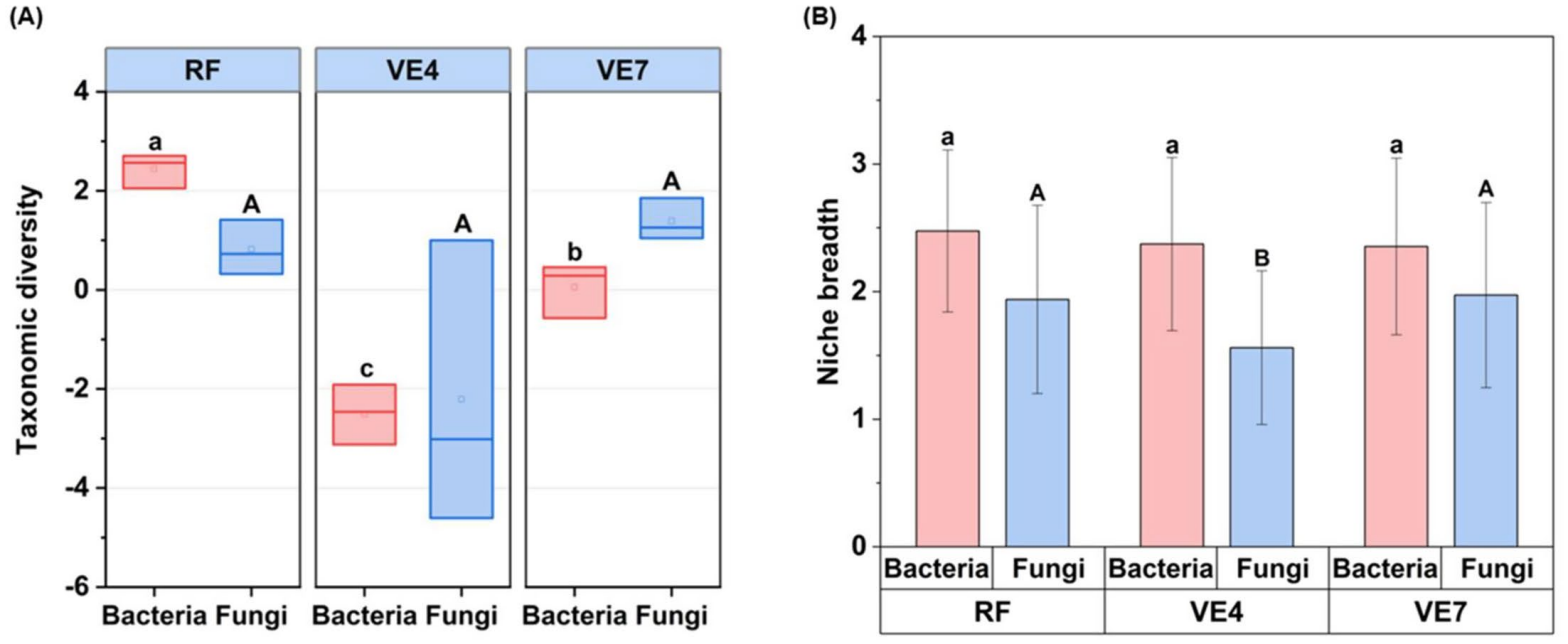
Microbial community diversity **(A)** and niche breadth **(B)** in a rice field (RF) and vegetable fields converted from the rice field for 4 years (VE4) and 7 years (VE7). Different lowercase letters above bars indicate significant differences among treatments for bacterial community, and different uppercase letters indicate significant differences for fungal community (*p* < 0.05). Error bars represent standard deviations (*n* = 3). Note: Six indices, Shannon index, Simpson index, Pilou index, coverage index, Heip index, and Pd index were used to reflect the taxonomic diversity in the analysis alpha diversity.

In terms of microbial diversity, land use conversion significantly altered the *β*-diversity of bacterial and fungal communities (Supplementary Figure S3), as well as the *α*-diversity of bacterial communities (Figure 2A). Community α-diversity was evaluated through six commonly used indices, namely Shannon, Simpson, Pielou, Coverage, Heip, and Pd (Supplementary Figure S4). The RF exhibited significantly higher taxonomic diversity compared to the VE4 and VE7 (*p* < 0.05). However, no significant differences were observed in the α-diversity of fungal communities among the different treatments (Figure 2A). Furthermore, changes in land use did not significantly reduce the ecological niche breadth of bacterial communities, while the fungal community niche breadth in VE4 was significantly reduced by 19.59% compared to RF (Figure 2B).

### 3.2 Variations soil microbial co-occurrence network

**Figure 3:**
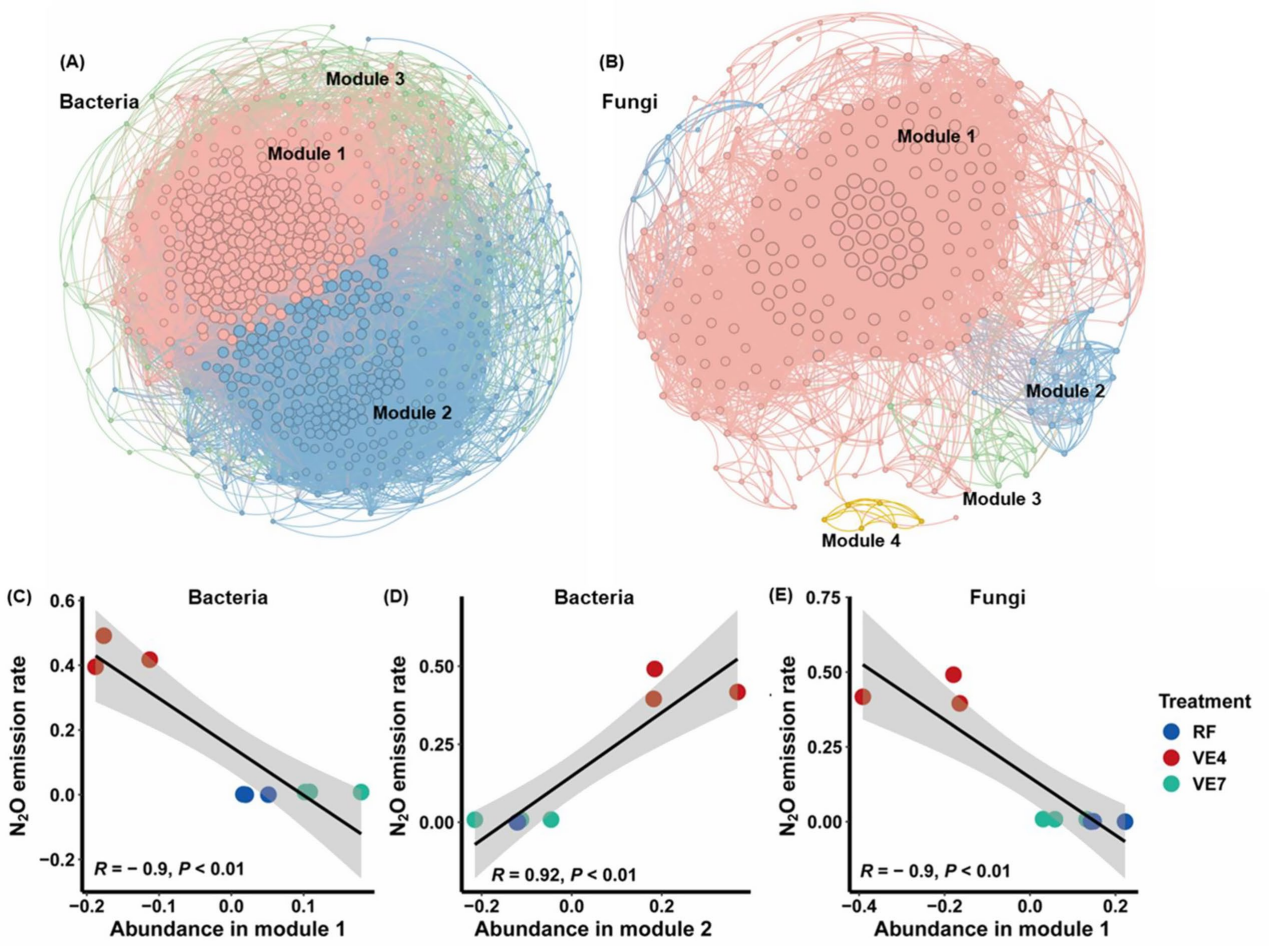
Network diagram with nodes colored according to each of the three main ecological clusters **(A,B)**. The modules in the networks are clusters of closely interconnected nodes. The size of each node reflects the number of connections (degree). The scatter diagram reflect regressions between the N_2_O emission rate and the relative abundance of the main bacteria in ecological clusters **(C–E)**.

The conversion of RF to vegetable cultivation significantly altered the structure of both bacterial and fungal co-occurrence networks (Figures 3A,B), as reflected in key topological characteristics and robustness. We identified three ecological clusters that exhibited significant co-occurrence relationships in the bacterial constructed network (Figure 3A; Supplementary Figure S5A). Within the co-occurrence network, *Sphaerobacter* and *Methylocaldum* were identified as core taxa (Supplementary Figure S6A). The network features of bacteria, such as the number of edges, positive connection edges, negative connection edges, number of vertices, average degree, and mean clustering coefficient were significantly lower in RF (*p* < 0.05). The number of positive connections under VE4 and VE7 land use types increased by 45.49 and 31.39%, respectively, compared to RF, while the number of negative connections increased by 59.57 and 39.39%, respectively (Supplementary Figure S7A). Under VE4 and VE7, the number of vertices was 1.24 and 1.15 times that of RF, respectively (Supplementary Figure S7B). Similarly, the average degree and mean clustering coefficient were 1.23 and 1.17 times, and 1.02 and 1.03 times higher than those under RF (Supplementary Figure S7C,D). But there was no significant difference in centralization betweenness between the land use type (Supplementary Figure S7E). The positive cohesion ranked as RF < VE7 < VE4 in bacterial co-occurrence networks (Supplementary Figure S7F). Moreover, the network robustness decreased significantly (*p* < 0.05) with increasing years since rice–vegetable conversion, being highest in the RF and lowest in the VF7 (Supplementary Figure S8A).

For the fungal network, four ecological clusters exhibiting significant co-occurrence relationships were identified (Figure 3B; Supplementary Figure S5B). *Gymnascella* and five other fungal genera were recognized as key nodes (Supplementary Figure S6B). The fungal network in the rice field (RF) exhibited significantly higher numbers of total edges, positive connection edges, and average degree as well as a greater mean clustering coefficient (*p* < 0.05), compared with those in the vegetable field soils (Supplementary Figures S9A,C,D). Conversely, the number of negative connection edges declined markedly with increasing years since conversion (*p* < 0.05). The number of vertices peaked in VF7, but declined by 11.44% and 27.49% in RF and VE4, respectively (Supplementary Figure S9B). In contrast, no significant variation was detected in the network’s centralization betweenness or positive cohesion across different land use types. Although VE4 and VE7 did not differ significantly in network robustness, both exhibited significantly greater robustness than the RF.

### 3.3 Key factors influencing N_2_O emissions

**Figure 4:**
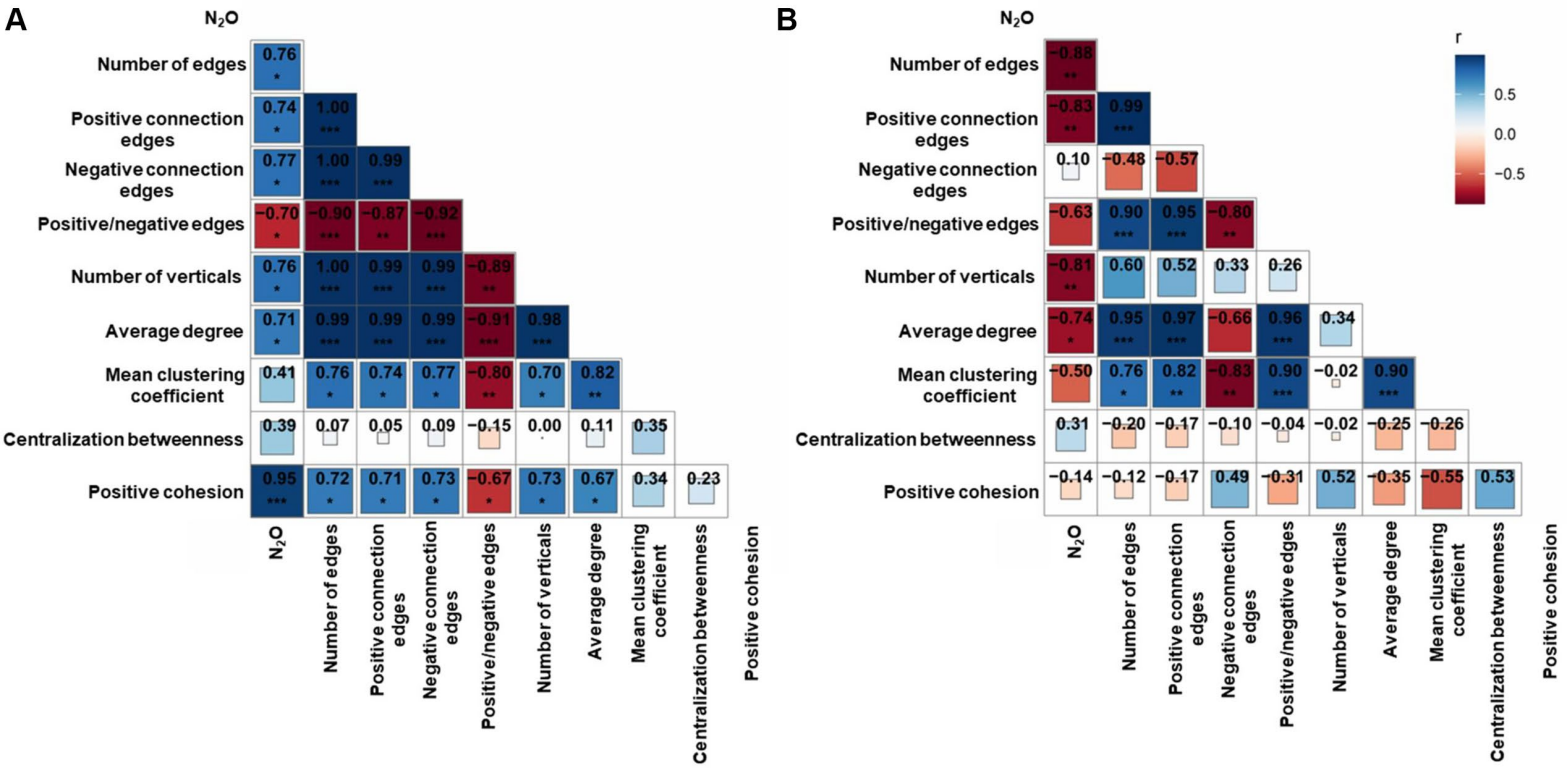
Pearson correlations between the N_2_O emission rate, bacterial network topological properties **(A)** and fungal network topological properties **(B)** in a rice field (RF) and vegetable fields converted from the rice field for 4 years (VE4) and 7 years (VE7). The color gradient and square denote Pearson’s correlation coefficients. Asterisks denote statistically significant differences at significance levels of *p < 0.05, **p < 0.01, and ***p < 0.001.

The relationship between microbial network characteristics and N_2_O emissions was evaluated by examining the topological characteristics of microbial (bacteria and fungi) networks. Notably, the number of edges, positive connection edges, negative connection edges, number of vertices, average degree and positive cohesion exhibited significant positive correlations with N_2_O emission rates in bacterial network (*p* < 0.05). Conversely, the ratio of positive/negative edges showed a significant negative correlation with N_2_O emission rates (*p* < 0.05; Figure 4A). Additionally, the number of edges, positive connection edges, number of vertices, and average degree in fungal network were significantly negatively correlated with N_2_O emission rates (*p* < 0.05; Figure 4B). Beyond network topological structure, the relative abundance of genera in different modules of the microbial network was also correlated with soil N_2_O emissions. Specifically, the relative abundance of genus in module 1 of the bacterial network and module 1 of the fungal network was significantly negatively correlated with N_2_O emissions (*p* < 0.05; Figures 3C,E). While the relative abundance of genus in module 2 of the bacterial network showed a significant positive correlation with N_2_O emissions (*p* < 0.05) (Figure 3D). The relative abundance of genus in other microbial network modules did not exhibit a significant correlation with soil N_2_O emissions (Supplementary Figure S10).

**Figure 5:**
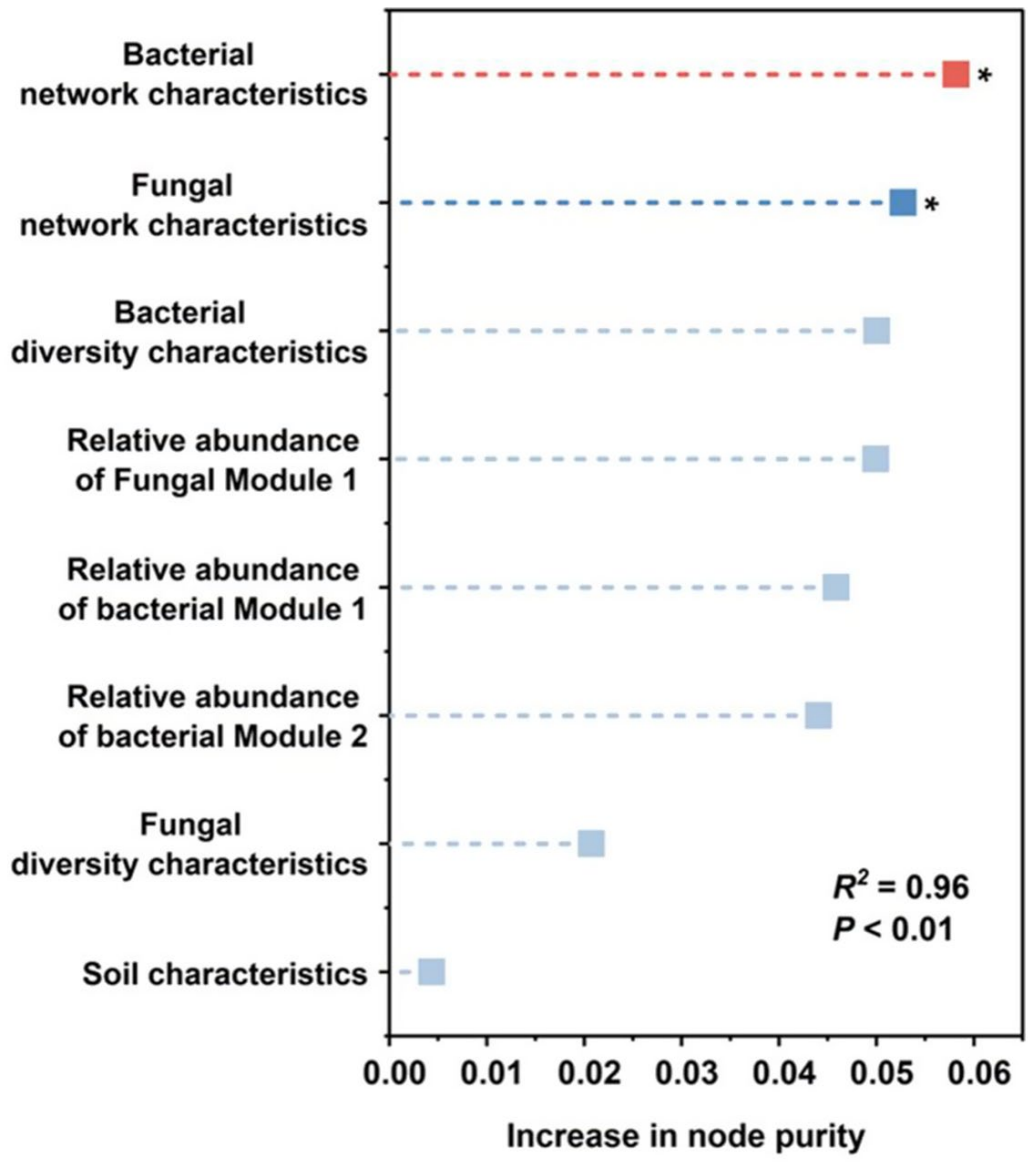
Potential driving factors influencing soil N_2_O emission rate variations during land-use conversion. The increase in node purity for each variable is used to estimate predictor importance, with higher values indicating greater importance. Asterisks denote statistically significant differences at significance levels of *p < 0.05. *R^2^* represents the explanatory power of the overall predictive model. The *p* value indicates the overall significance of the model.

To further identify the key factors influencing N_2_O emissions, a Random Forest analysis was conducted using environmental variables (Supplementary Figure S11), taxonomic diversity, the relative abundance of genus in different modules, and network characteristics as predictors (Supplementary Figure S12). The explanatory power of the model reached 96% (*p* < 0.05). The analysis revealed that bacterial network characteristics and fungal network characteristics were the most significant predictors of N_2_O emissions (Figure 5). Furthermore, the analysis indicated that the diversity characteristics of the bacterial community and the relative abundance of genera in module 1 of the fungal network play a relatively important role in regulating N_2_O emissions, although this did not reach statistical significance. Among these factors, soil properties were identified as the weakest driver.

## 4 Discussion

### 4.1 Land use change alters soil microbial community composition

Increasing land use intensity and N management input generally enhance N_2_O emission rates (Uwiragiye et al., 2024). For instance, the conversion of natural forests to croplands typically stimulates soil N_2_O release (Zheng et al., 2020; Xiao et al., 2025). Regions showing pronounced increases in N_2_O emissions following land use change are often characterized by high levels of reactive nitrogen inputs (Ma J. Y. et al., 2025). In our specific land use conversion system, we likewise observed a significant increase in soil N_2_O emissions following the conversion of rice fields to vegetable cultivation. This pattern is consistent with a study conducted in Jiangxi Province, China, where the conversion of double-cropping rice fields to vegetable cultivation increased the soil N_2_O emission rate by 14.12 times (Yuan et al., 2016). It is noteworthy that although the elevated trend of N_2_O fluxes in vegetable fields relative to the rice field persisted across different cultivation durations, the N_2_O flux in the VE7 soil was significantly lower than that in VE4. This suggests that under long-term vegetable cultivation, the microbial processes in VE7 soil may have reached a new steady or adaptive state (Ji et al., 2021). Such a state could be attributed to shifts in microbial community composition (Shen et al., 2021), changes in nutrient availability (Li L. Z. et al., 2022), strengthened microbial interactions (Lin et al., 2025; see also Section 4.3 for further discussion on their role in N_2_O dynamics), or altered niche breadth.

The conversion from rice to vegetable fields alters microbial community composition. In the RF soil, the three most abundant bacterial genera were mainly decomposers with strong capacities for organic carbon degradation but showed a complete or partial absence of denitrification-related functional genes (Zhang et al., 2021; Yessentayeva et al., 2024). As a representative, *Candidatus_Solibacter* encodes diverse carbohydrate-active enzymes and phenol oxidases, which enable it to mineralize plant residues and humic substances effectively (Mastny et al., 2021). By contrast, continuous vegetable cultivation supported a bacterial community dominated by *Gemmatimonas* and *Bacillus*. These microorganisms are key participants in denitrification (Liu et al., 2022; Huang et al., 2025), likely contributing to the increased N_2_O emissions detected in vegetable soils. Moreover, *Mortierella* was the most abundant fungal genus in RF soil. Jiang et al. (2021) similarly reported that during the milk stage of rice, the peak relative abundance of *Mortierella* could reach 50%. Analogous to the bacterial communities, the dominant fungal genera in VF4 and VF7 were largely similar after rice-to-vegetable conversion. Although they have been rarely studied, these fungi are presumed to contribute to soil organic matter decomposition (Piontelli et al., 1981), highlighting the need for further research. As for community diversity, microbial community diversity exhibited consistent patterns for both bacteria and fungi. It was highest in RF soils, decreased after 4 years of conversion to vegetable fields, and increased after 7 years. This indicates that rice-to-vegetable conversion may initially disrupt microbial community function, but over time, communities tend to stabilize into a new steady state. These trends align with the temporal pattern of soil N_2_O emissions, suggesting that changes in microbial diversity may play a key role in regulating N_2_O fluxes (Shen et al., 2021). In addition, after 4 years of rice-to-vegetable conversion, the fungal niche breadth significantly decreased, likely due to enhanced cultivation intensity that imposed stronger environmental filtering (Zhou and Wu, 2021). The narrowing of fungal niche breadth may have altered the balance between C decomposition and N transformation (Ren et al., 2023). Specifically, the loss of fungal taxa with high capacities for organic matter turnover could have reduced the C substrates available to denitrifying bacteria, thereby promoting incomplete denitrification and indirectly enhancing N_2_O emissions (Chen Z. et al., 2021). The findings highlight the pivotal role of fungi in maintaining N cycling efficiency and mitigating N_2_O emissions during land use transitions.

### 4.2 Soil microbial co-occurrence network variations

The characteristic of the microbial co-occurrence network was significantly influenced by the conversion of rice to vegetable fields. In the bacterial network, three ecological clusters with significant co-occurrence relationships were identified, while the fungal network contained four clusters. Notably, not all microbial groups were significantly correlated with soil N_2_O emissions, but rather, specific microbial groups within certain modules. In agricultural ecosystems, specific environmental selection pressures may lead to the emergence of subsets of beneficial and detrimental microbial groups within the network (Fan et al., 2019), which have differential impacts on nitrogen cycling processes (Yu et al., 2024). As a result, only microbial groups within specific modules showed significant correlations with soil N_2_O emissions (Jones et al., 2022). However, the keystone taxa within the networks exhibited weak direct associations with soil N_2_O emissions (Supplementary Figure S13), suggesting that they may exert their effects by driving module-level metabolic processes rather than by directly regulating N_2_O fluxes. For example, *Sphaerobacter* is capable of efficiently degrading cellulose (Hou et al., 2025), whereas *Methylocaldum* has been shown to influence energy metabolism and thereby play a key role in N transformation processes (Chu et al., 2025).

Following the conversion of rice fields to vegetable cultivation, management practices were profoundly altered, leading to greater fluctuations in soil physicochemical properties (Wu et al., 2023). The bacterial community responded rapidly to these environmental perturbations (Wang et al., 2024), reorganizing its community composition and forming new interspecific associations. This dynamic adjustment increased the number of correlations among nodes within the network, resulting in a temporary rise in overall network complexity (Huang et al., 2024). However, this apparent complexity largely reflects short-term “stress-induced linkages” rather than stable, functionally coherent relationships. As environmental conditions continue to change, bacterial communities may establish new species interactions through rapid adaptive mechanisms (Sun et al., 2025). Such interactions are often fragile and lack sufficient functional redundancy. Low redundancy implies that when the system experiences external disturbances, there are limited alternative pathways or compensatory functions available, thereby reducing the overall robustness and resilience of the bacterial network (Allison and Martiny, 2008). Compared with bacteria, fungal communities generally exhibit a higher degree of functional redundancy (Zhou et al., 2022), meaning that even if certain taxa are functionally impaired, others can compensate to maintain essential ecological functions (Zeng et al., 2023; Moreira et al., 2025). Land use changes may reduce the complexity of fungal networks, but this does not necessarily imply a loss of function. Instead, fungi may optimize resource use efficiency and adaptive capacity by simplifying their interaction networks (Yang et al., 2022). In dynamic environments, simplified fungal networks can be more efficient in resource allocation than highly complex ones. By reducing unnecessary functional redundancy, fungal communities may direct their activity toward essential ecosystem functions, including the decomposition of organic substrates and nitrogen cycling (Bao et al., 2024). This adaptive strategy enhances the robustness of fungal networks, enabling them to sustain ecosystem functionality under environmental disturbances (Xiong et al., 2024). Our results are not fully consistent with previous findings. Cornell et al. (2023) reported that within 19 months of converting grassland to wheat cropland, both the microbial network’s stability and complexity increased simultaneously. The discrepancy between our results and theirs may stem from differences in land use conversion pathways. In the previous study, the system transitioned from natural grassland to cultivated land, whereas in our study, the conversion was from a flooded environment to upland conditions.

### 4.3 Mechanistic integration: linking land use change, microbial networks, and N_2_O emissions

The conversion of rice fields to vegetable cultivation may fundamentally alter bacterial co-occurrence patterns, thereby reshaping soil N transformation pathways and leading to increased N_2_O emissions. As bacterial networks become more complex yet fragile, the stability of bacterial cooperation may decline (Yonatan et al., 2022), potentially disrupting the delicate balance of N transformation processes. In particular, alterations in the connectivity of denitrifying bacteria may hinder the complete reduction of N_2_O to N_2_, resulting in the accumulation of the intermediate product (Carr et al., 2025). Furthermore, under fluctuating environmental conditions, rapid community turnover may favor opportunistic bacterial species with high N_2_O producing but limited reducing capacities, thereby further enhancing N_2_O emissions (Shakoor et al., 2025).

Unlike bacterial networks, this study found that a decline in fungal network complexity accompanied by increased robustness was associated with a significant rise in soil N_2_O emissions. This finding suggests that during the conversion of rice fields to vegetable cultivation, the ecological “integration” of fungal networks may enhance system stability, yet concurrently favor functional groups that promote N_2_O production rather than its reduction. Such simplified but more robust fungal networks are typically dominated by a few stress-tolerant or resource-efficient taxa (Zhang et al., 2025). These fungi are mostly saprotrophic or opportunistic species that can accelerate organic matter decomposition and N mineralization (Camenzind et al., 2024), thereby increasing the availability of inorganic N and providing a continuous substrate supply for bacterial denitrification. From a functional perspective, FUNGuild-based annotation revealed that saprotrophic fungi were dominant in VE4 (Supplementary Figure S14), supporting the view that fungal networks may primarily influence denitrification by regulating organic matter decomposition.

The disruption of the C-N balance under such conditions may lead to sustained N_2_O emissions (Zhu et al., 2023). Moreover, the decline in fungal network complexity weakened its regulatory role in the coupling of carbon and nitrogen cycles (Booth et al., 2019). In complex networks, diverse fungal taxa interact with bacteria through competitive or mutualistic relationships, forming multilayered linkages that buffer N loss and maintain soil redox balance (Wagg et al., 2019). Once the network is simplified, such ecological regulation diminishes—fungal N immobilization becomes less effective, while incomplete bacterial denitrification dominates, leading to enhanced N_2_O accumulation. Thus, the observed “robustness” may, in fact, represent a stable state characterized by persistently high N_2_O emissions.

It is noteworthy that in both bacterial and fungal networks, the ratio of positive to negative connections exhibited a strong negative association with N_2_O emissions. Networks characterized by lower positive/negative connection ratios typically exhibit intensified internal competition (Ma et al., 2020). This finding further suggests that weakened microbial cooperation and enhanced competitive interactions disrupt the functional coordination among N-cycling guilds, thereby increasing N_2_O emissions. The recovery of the positive-to-negative connection ratio in VE7 compared with their lower levels in VE4 suggests that microbial processes in VE7 soils may be, or are in the process of, reaching a new steady state. Finally, the random forest analysis reaffirmed that microbial network features were the most influential predictors of N_2_O fluxes, surpassing traditional soil physicochemical variables. Consistently, studies in various ecosystems have also highlighted the pivotal role of microbial network architecture in regulating soil N_2_O emissions (Jameson et al., 2023; Qiu et al., 2023). Furthermore, no significant associations were detected between integrated soil properties and microbial network characteristics (Supplementary Figure S15). Consequently, the indirect regulatory effect of integrated soil physicochemical properties on N_2_O emissions mediated through microbial network features appears to be relatively weak.

Taken together, a potential mechanistic framework emerges: the conversion of rice fields to vegetable cultivation reshapes the composition and co-occurrence architecture of microbial communities. Within this context, microbial networks act as key mediators that translate environmental perturbations into changes in ecosystem process rates. The contrasting responses of bacterial and fungal networks highlight their complementary ecological roles—bacterial networks are closely linked to denitrification, whereas fungal networks may indirectly regulate N_2_O dynamics by influencing soil carbon turnover and microhabitat accessibility. Nevertheless, it should be emphasized that our findings are mainly indicative of microbial responses to site-specific differences in the potential denitrification-derived N_2_O emissions, rather than direct responses to actual field emissions.

### 4.4 Limitations and future directions

Field N_2_O emissions originate from multiple processes, including nitrification, denitrification, and their coupled pathways under fluctuating redox conditions (e.g., nitrifier denitrification) (Zhu G. B. et al., 2025). The measurement approach used in this study represents only the potential N_2_O production associated with denitrification under controlled anaerobic conditions. Consequently, the N_2_O emission results presented here should be interpreted as a process-based indicator that aids in explaining the spatial variability of field N_2_O emissions and provides mechanistic insight into the role of denitrification in shaping field-scale N_2_O emission patterns, rather than as a direct quantitative prediction of in situ N_2_O fluxes. Besides, the reshaping of microbial networks induced by land use change underscores the urgent need for integrated management strategies that account for microbial dynamics when mitigating N_2_O emissions. Emerging evidence suggests that interventions such as crop diversification and the application of organic amendments can enhance nutrient cycling efficiency in agricultural soils while simultaneously reducing greenhouse gas emissions (Li et al., 2024). A deeper understanding of how these practices influence microbial interactions and network architecture in regulating N_2_O fluxes will enable more precise soil management strategies that balance agricultural productivity with environmental sustainability. Although our study reveals strong correlations between microbial network properties and N_2_O emissions, the limited sample size may reduce the statistical power and thus increase the risk of type II errors, especially in microbial co-occurrence network analyses. Therefore, the results of this study should be interpreted primarily as indicative of potential ecological patterns. Future studies could verify the robustness of our findings by increasing the sample size or incorporating multi-scale sampling approaches. Manipulative experiments targeting specific network modules or taxa would clarify their functional roles. Long-term monitoring across diverse soil types and climatic regions is also needed to generalize the observed patterns. Furthermore, integrating metagenomic or transcriptomic analyses could link network structure to gene expression, providing deeper mechanistic insight into microbial contributions to N_2_O dynamics.

## 5 Conclusion

Overall, our findings demonstrate that converting rice fields to vegetable cultivation markedly alters microbial community composition and stimulates the potential denitrification-derived N_2_O emissions. Specifically, compared with the original rice soils, bacterial co-occurrence networks in vegetable soils exhibited a greater number of edges, higher connectivity, and lower robustness. In contrast, fungal networks showed fewer edges, reduced connectivity, and enhanced robustness. With increasing cultivation duration, the number of edges and the degree of connectivity in bacterial networks significantly decreased, whereas those in fungal networks increased. Moreover, microbial community diversity exhibited a pattern of initial decline followed by recovery after land use conversion. Collectively, our results suggest that microbial network characteristics serve as the most critical predictors of the potential denitrification-derived N_2_O emissions during rice to vegetable conversion. This finding highlights that microbial communities are not merely passive responders to environmental change but actively mediate biogeochemical processes through their interaction networks. Given the ongoing global shifts in land use patterns, integrating microbial network features into predictive models of soil greenhouse gas emissions will be essential for improving the accuracy of N_2_O emission assessments under future land use change scenarios.

## Data Availability

The datasets presented in this study can be found in online repositories. The names of the repository/repositories and accession number(s) can be found in the article/Supplementary material.

## Footnotes

1
https://gephi.org/
