# Supplementary material for: Shifted microbial network characteristics govern soil N2O emission following paddy-to-vegetable land conversion
Source: Front Microbiol. 2026 Jan 29;17:1750894. doi: 10.3389/fmicb.2026.1750894 (PMC12894308; doi:10.3389/fmicb.2026.1750894)
Supplement: Supplementary file 1 [file Data_Sheet_1.docx]

**Supporting Information**

Title: **Shifts in microbial network characteristics govern soil N_2_O emission following paddy-to-vegetable land conversion**

Authors: Chenglin Li ^a, b, c^, Ziqun Zhou^a^, Xin Chen^a^, Quan Tang^d^, Qingbi Zhang^a*^, Jieshi Tang^a, c*^

Address:

^a^ Environmental Health Effects and Risk Assessment Key Laboratory of Luzhou, School of Public Health, Southwest Medical University, Luzhou 646000, China

^b^ State Key Laboratory of Soil and Sustainable Agriculture, Institute of Soil Science, Chinese Academy of Sciences, Nanjing 210008, China

^c^ College of Life Sciences, Shihezi University, Shihezi 832003, China

^d^ Key Laboratory of Arable Land Quality Monitoring and Evaluation, Ministry of Agriculture and Rural Affairs, Yangzhou University, Yangzhou 225009, China

*: Corresponding Author email: jieshi_tang@126.com; swmutgyx@163.com

**Numbers of pages: 20**

**Number of Tables: 2**

**Numbers of Figures: 15**

**Bioinformatics analysis process**

Raw fastq files were quality-filtered using Trimmomatic and merged with FLASH. (Wei et al., 2022). The sequencing data were further refined based on quality scores using Quantitative Insights into Microbial Ecology (QIIME) (Caporaso et al., 2010). Operational taxonomic units (OTUs) were clustered at a 97% similarity threshold using the UPARSE pipeline (version 11, http://drive5.com/uparse/), employing a novel ‘greedy’ algorithm for simultaneous chimera filtering and OTU clustering. Taxonomic classification of each 16S rRNA and ITS gene sequence was performed using the RDP Classifier algorithm, referencing the Silva 132 and Unite 9.0 databases (Zhu et al., 2024), with a confidence threshold set at 0.7. Fungal trophic modes were analyzed using the FUNGuild database. Finally, the raw amplicon data have been deposited at the Beijing Institute of Genomics Data Center, Chinese Academy of Sciences (http://bigd.big.ac.cn/gsa), with accession number CRA005689 and CRA032792.

|  | Total C | Total N | DOC | CEC | pH | NO_3_^-^-N |
| --- | --- | --- | --- | --- | --- | --- |
| Samples | (g kg^−1^) | (g kg^−1^) | (mg kg^−1^) | (cmol kg^−1^) |  | (mg kg^−1^) |
| RF | 26.67±5.31 a | 2.60±0.64 a | 217.63±2.43 a | 23.73±0.36 a | 6.16±0.02 a | 0.39±0.04 c |
| VE4 | 12.80±0.10 b | 1.27±0.10 b | 82.65±2.80 c | 19.46±0.20 b | 5.88±0.03 b | 3.69±0.12 b |
| VE7 | 15.07±0.06 b | 1.94±0.11 a | 89.20±1.59 b | 19.96±0.44 b | 4.19±0.01 c | 32.92±0.84 a |

**Table S1** Soil properties of the rice and vegetable soils

Data are expressed as the mean ± standard deviation (*n* = 3). The different letters in the same column indicate significant differences among the treatments (*P* < 0.05).

| Specificity | Primer | Sequence (5’-3’) | PCR program | Reference |
| --- | --- | --- | --- | --- |
| ***16S*** | 515F | GTGCCAGCMGCCGCGG | 3 min of denaturation at 95 °C, 27 cycles of 30 s at 95 °C, 30 s of annealing at 55 °C, and 45 s of elongation at 72 °C | ([Dong et al., 2024](#_ENREF_2); [Zhu et al., 2024](#_ENREF_5)) |
|  | 907R | CCGTCAATTCMTTTRAGTTT |  |  |
| ***ITS*** | ITS1F | CTTGGTCATTTAGAGGAAGTAA | 5 min of denaturation at 95 °C, 35 cycles of 30 s at 95 °C, 30 s of annealing at 58 °C, and 1 min of elongation at 72 °C | ([Wei et al., 2022](#_ENREF_3); [Yu et al., 2025](#_ENREF_4)) |
|  | ITS2R | GCTGCGTTCTTCATCGATGC |  |  |

**Table S2** Primers and PCR conditions used in this study


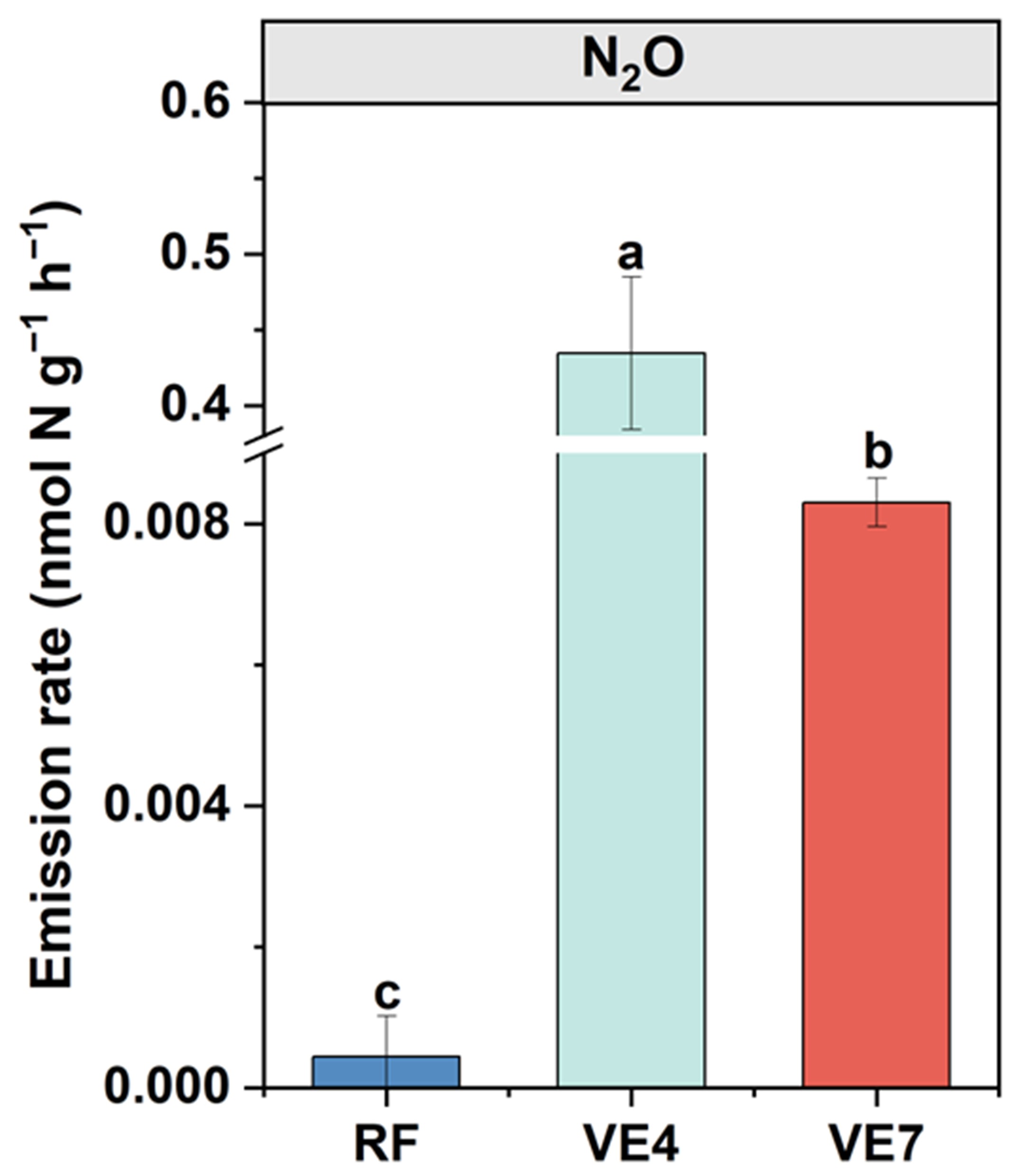


Figure S1. Changes in soil N_2_O emission rates following conversion from paddy (RF) to vegetable land for 4 (VE4) and 7 (VE7) years. Values are the means with standard deviations (*n* = 3). The different letters above the columns indicate significant differences among the treatments (*P* < 0.05).


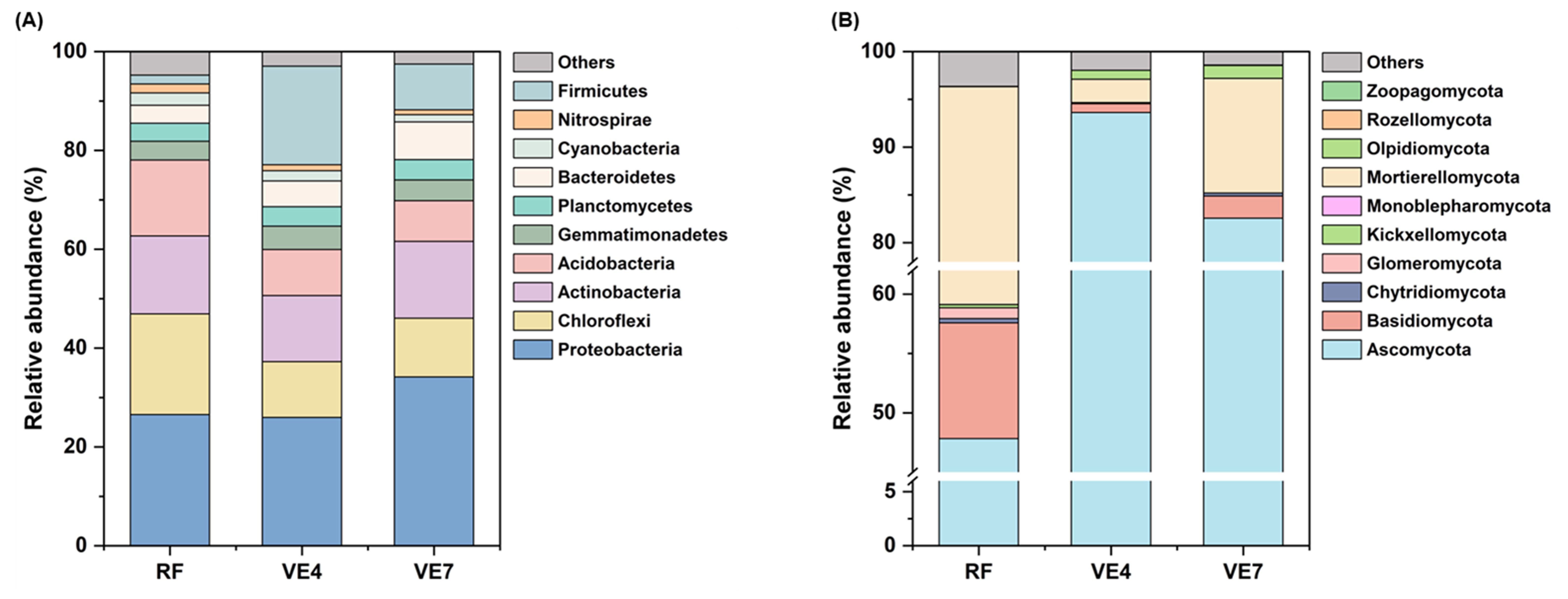


Figure S2. The relative abundance of dominant bacterial (A) and fungal (B) phyla in a rice field (RF) and vegetable fields converted from the rice field for 4 years (VE4) and 7 years (VE7).


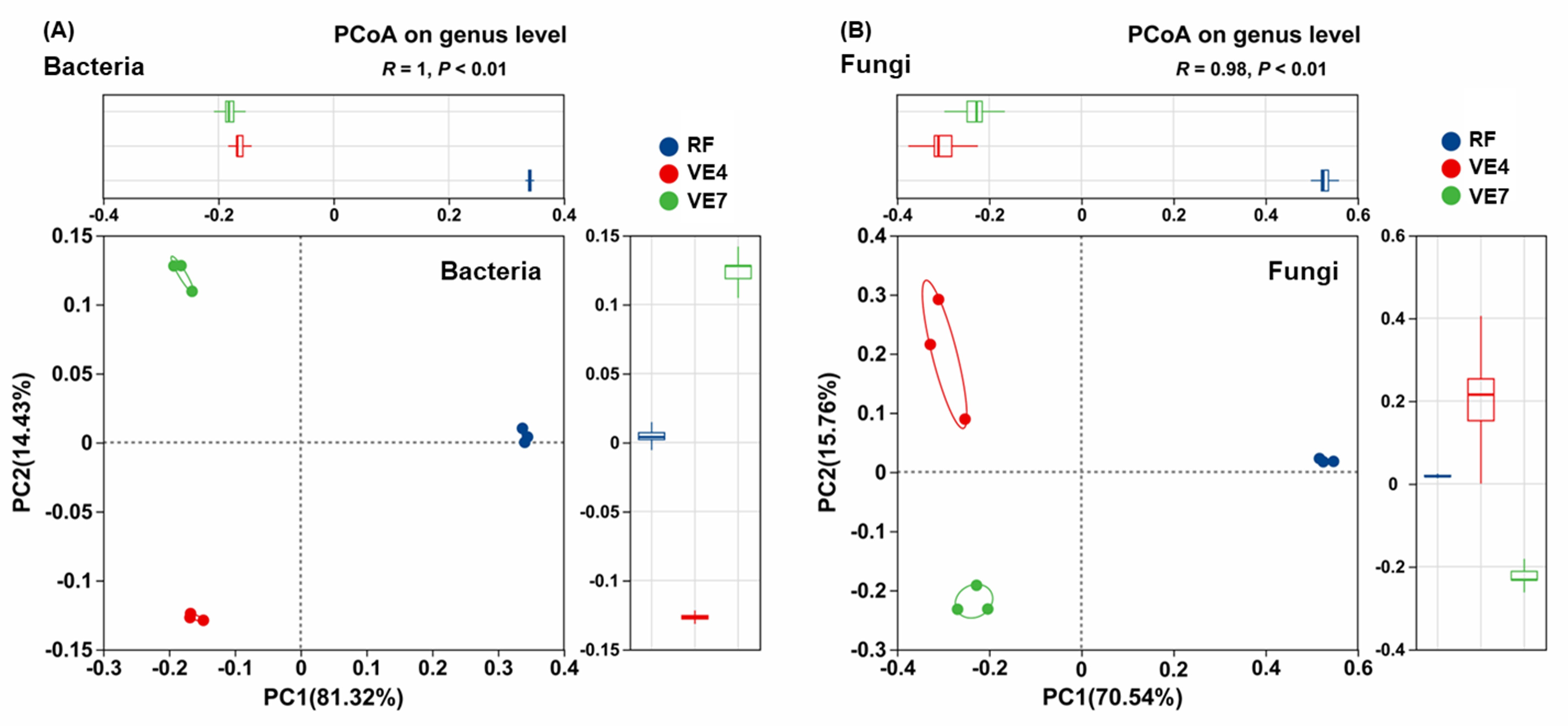


Figure S3. Changes in soil bacterial (A) and fungal (B) community composition following conversion from paddy (RF) to vegetable land for 4 (VE4) and 7 (VE7) years. Note: The values of *P* and *R* in the graph were obtained by ANOSIM test.


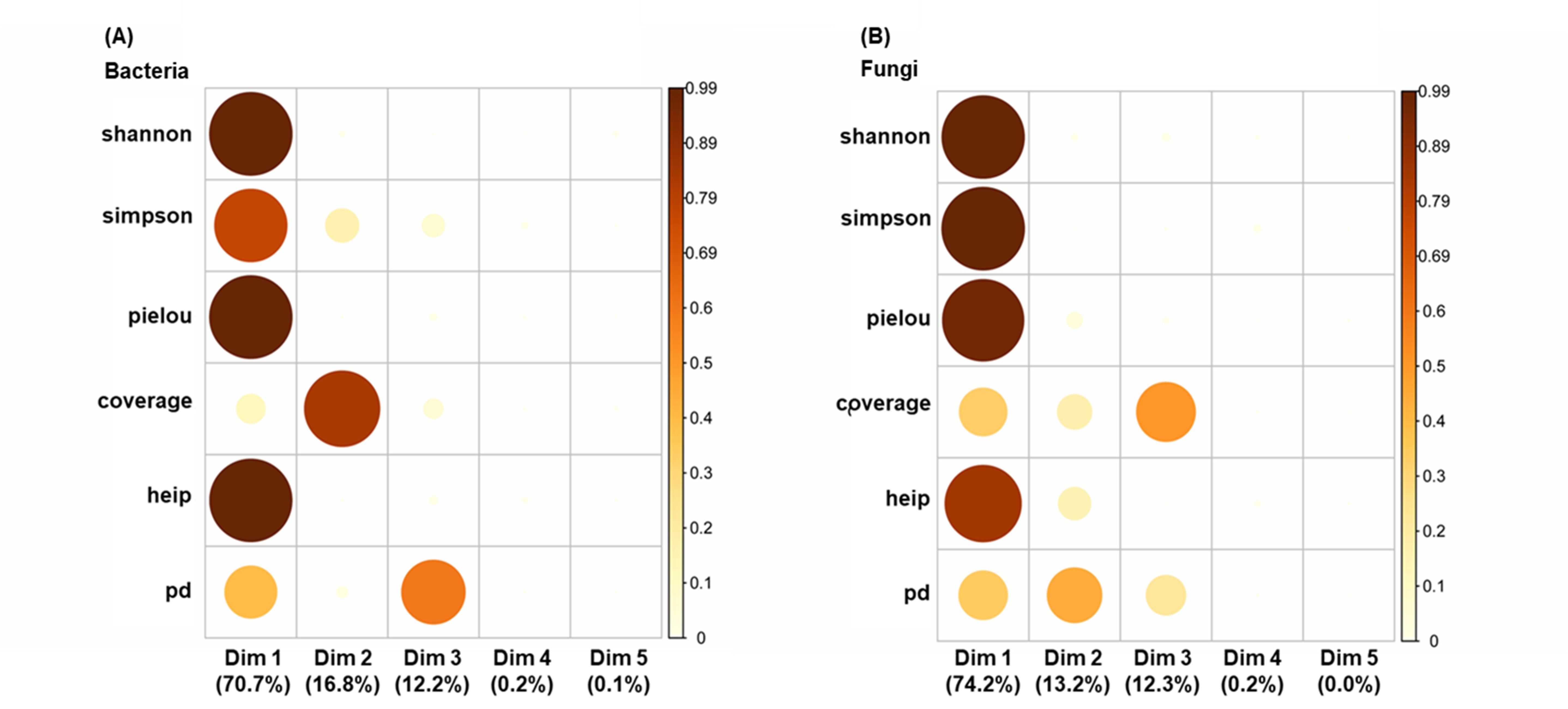


Figure S4. The explanatory ability of different principal components for bacterial (A) and fungal (B) community diversity obtained from PCA analysis. The red gradient represents the degree of explanatory ability, where darker colors denote higher explanatory strength.


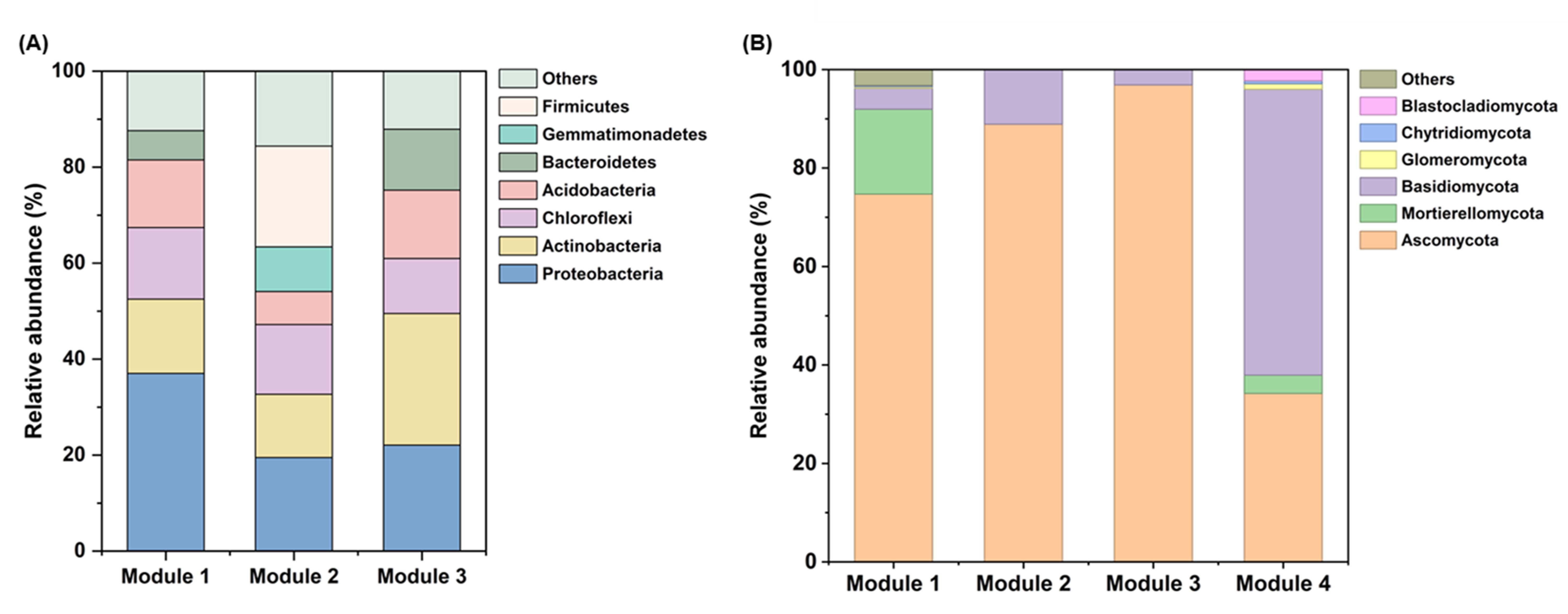


Figure S5. The relative abundance of dominant bacterial (A) and fungal (B) phyla in different ecological clusters across a rice field (RF) and vegetable fields converted from the rice field for 4 years (VE4) and 7 years (VE7).


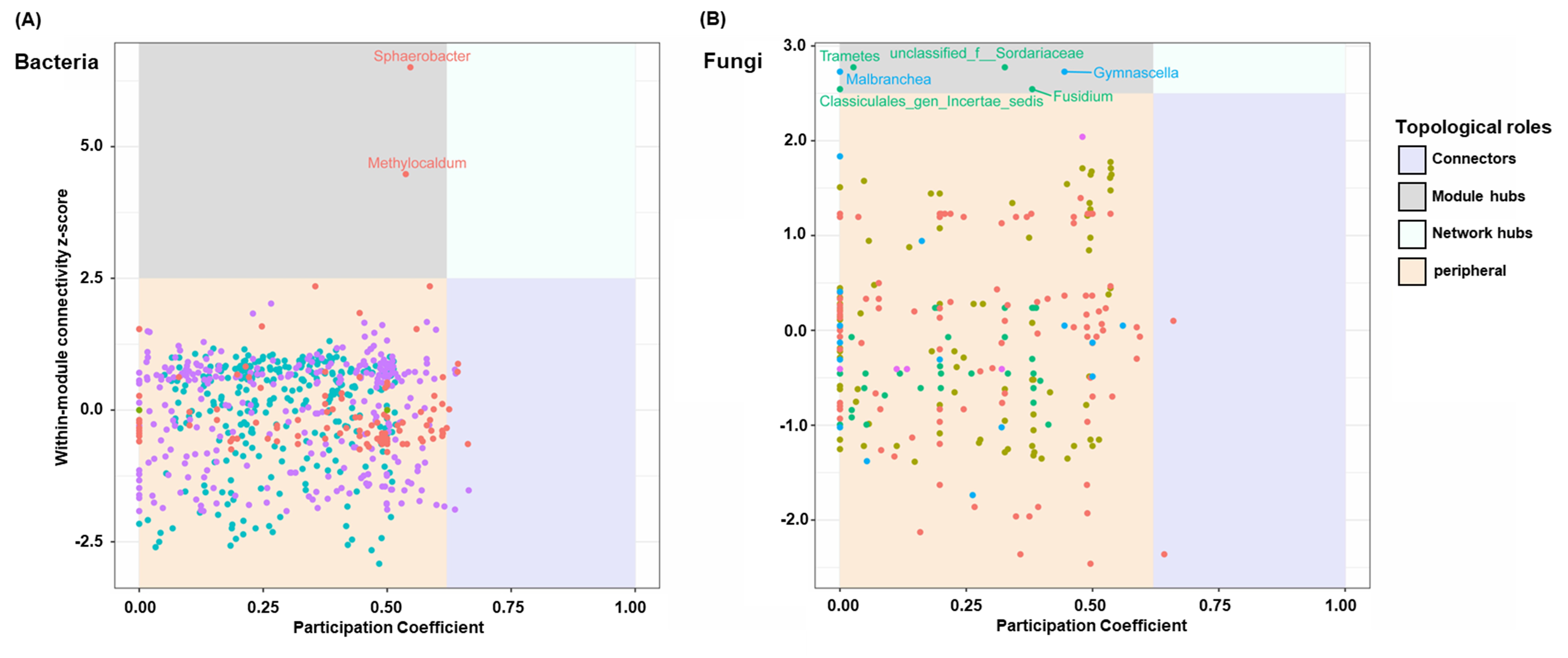
Figure S6. Distribution of bacterial (A) and fungal (B) genera based on their network roles. Nodes in the network are classified as peripherals, modular hubs, network hubs or connectors depending on their role in the network.


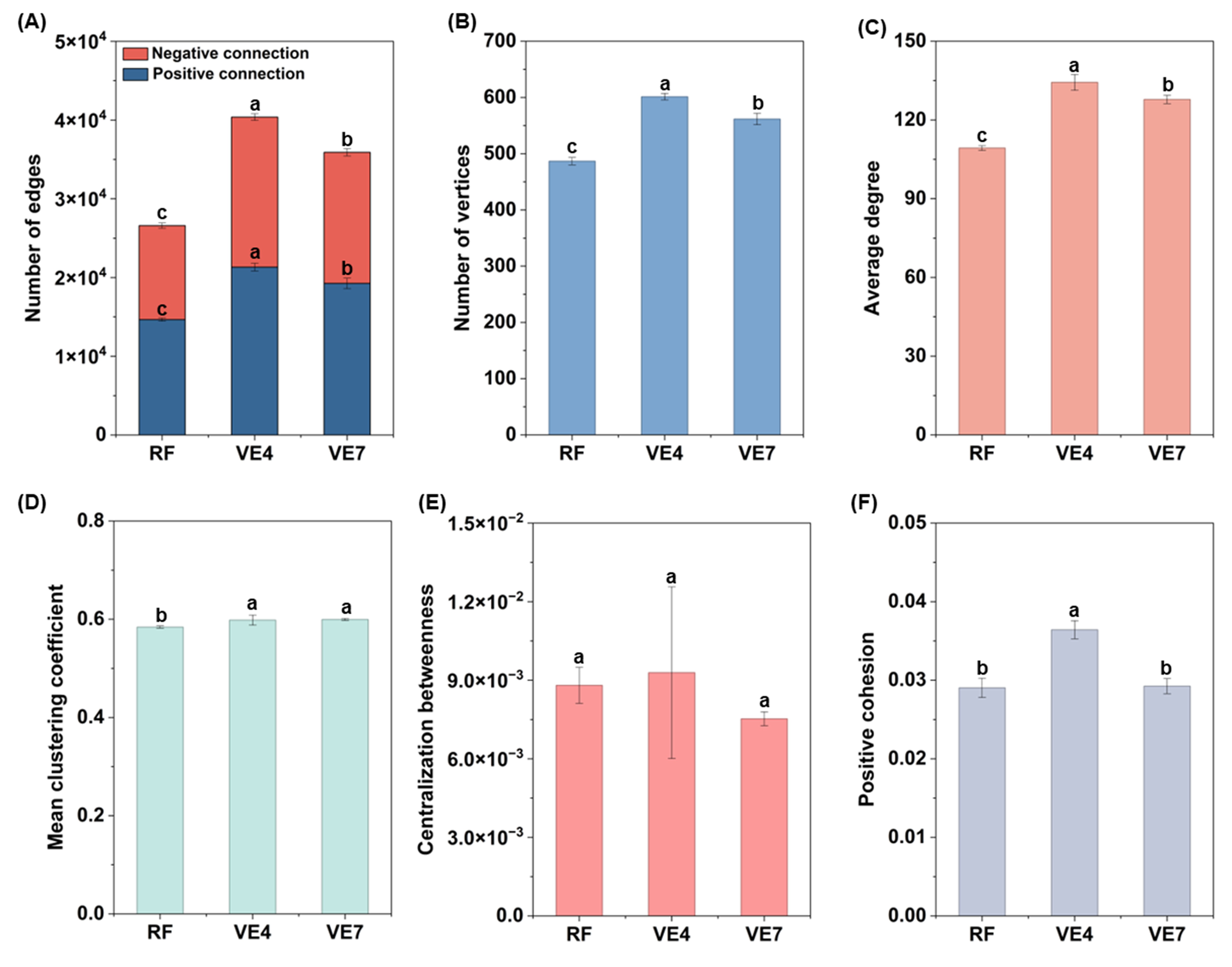
Figure S7. Topological features of bacterial co-occurrence networks in a rice field (RF) and vegetable fields converted from the rice field for 4 years (VE4) and 7 years (VE7). Values are the means with standard deviations (*n* = 3). The different letters above the columns indicate significant differences among the treatments (*P* < 0.05).


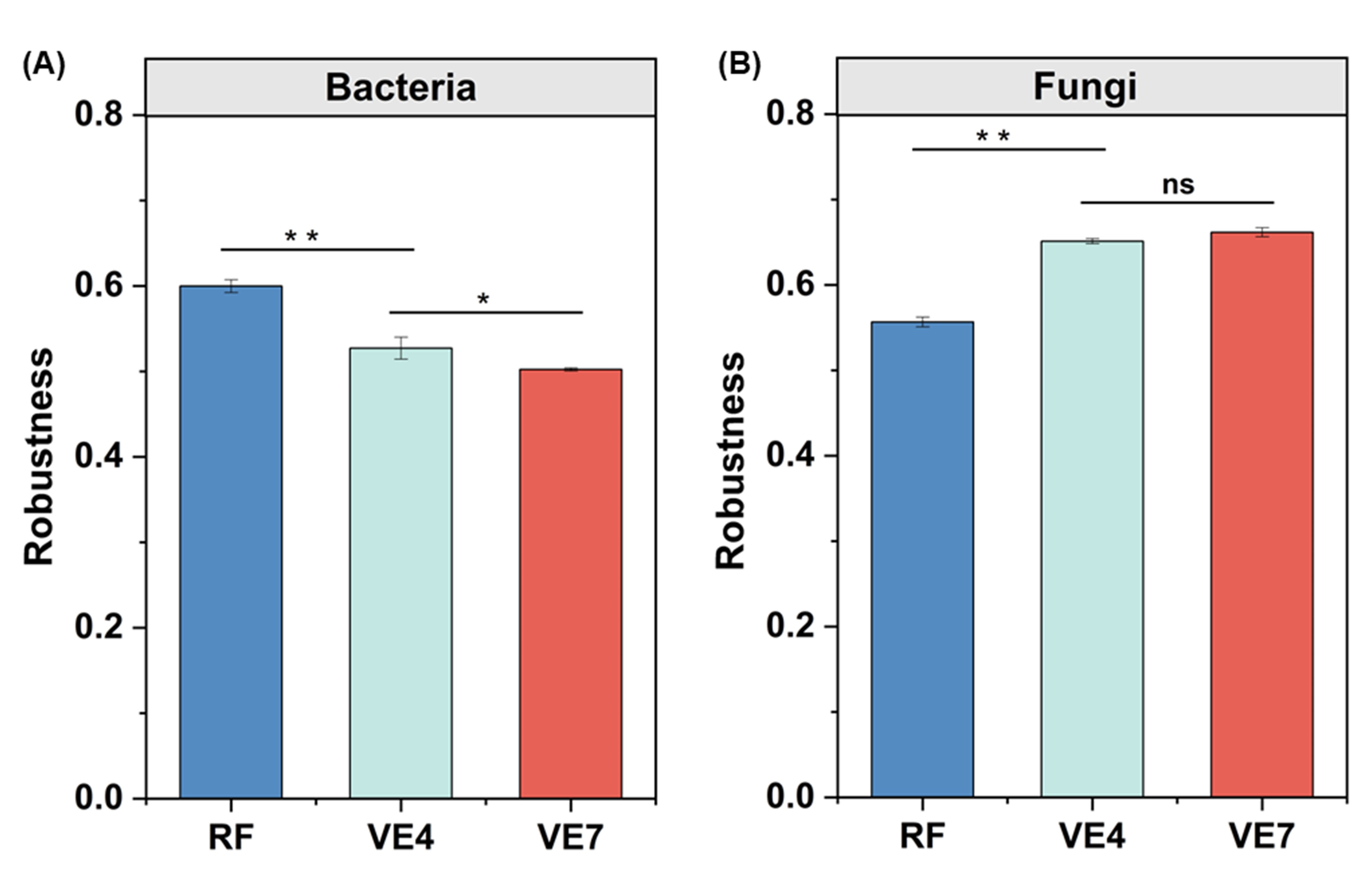
Figure S8. Robustness measured by the proportion of taxa retained after removing five hub modules from bacterial (A) and fungal (B) networks in a rice field (RF) and vegetable fields converted from the rice field for 4 years (VE4) and 7 years (VE7). Each error bar corresponds to the standard deviation of 100 repetitions of the simulation. Asterisks denote statistically significant differences at significance levels of * *P* < 0.05 and ** *P* < 0.01.


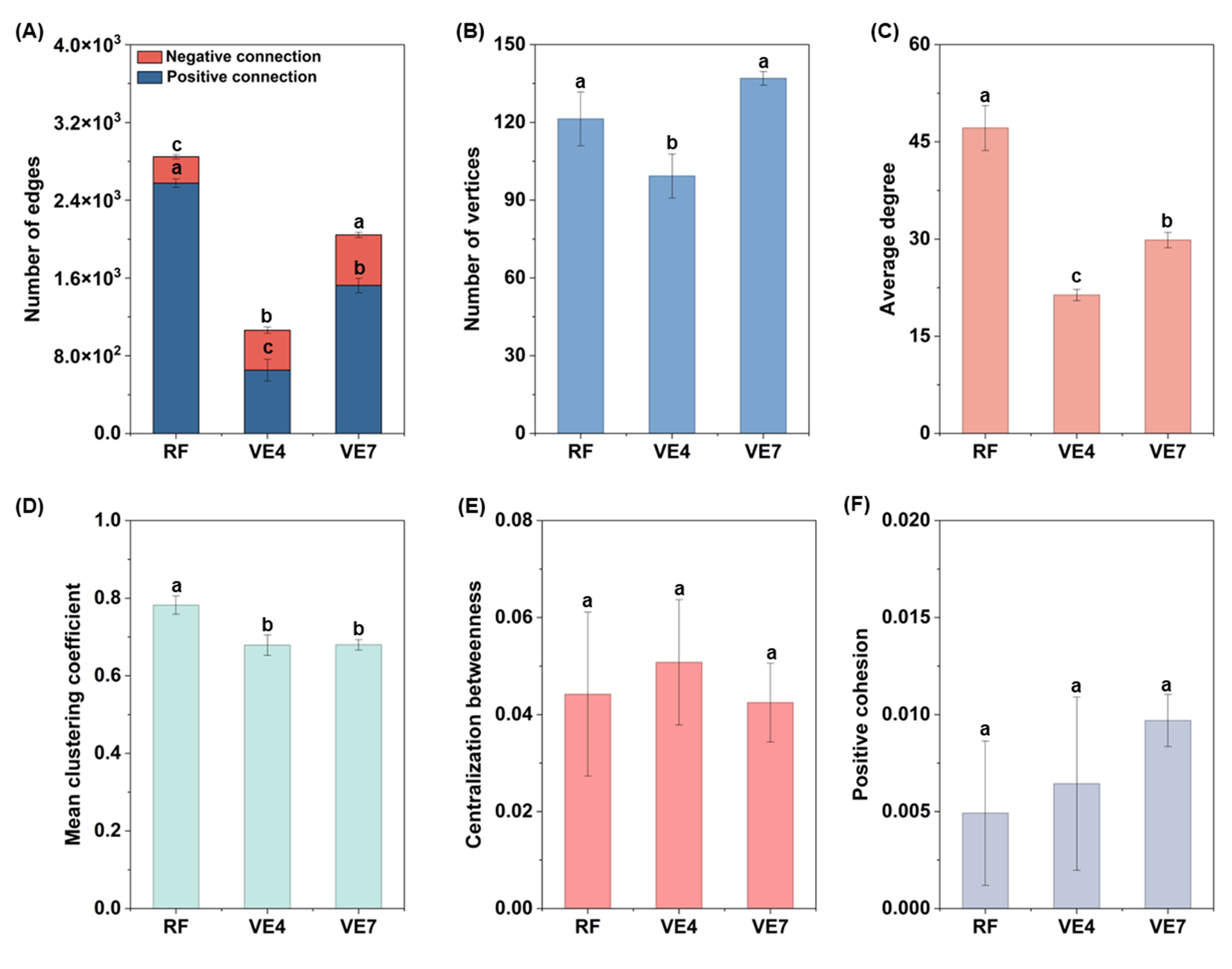
Figure S9. Topological features of fungal co-occurrence networks in a rice field (RF) and vegetable fields converted from the rice field for 4 years (VE4) and 7 years (VE7). Values are the means with standard deviations (*n* = 3). The different letters above the columns indicate significant differences among the treatments (*P* < 0.05).


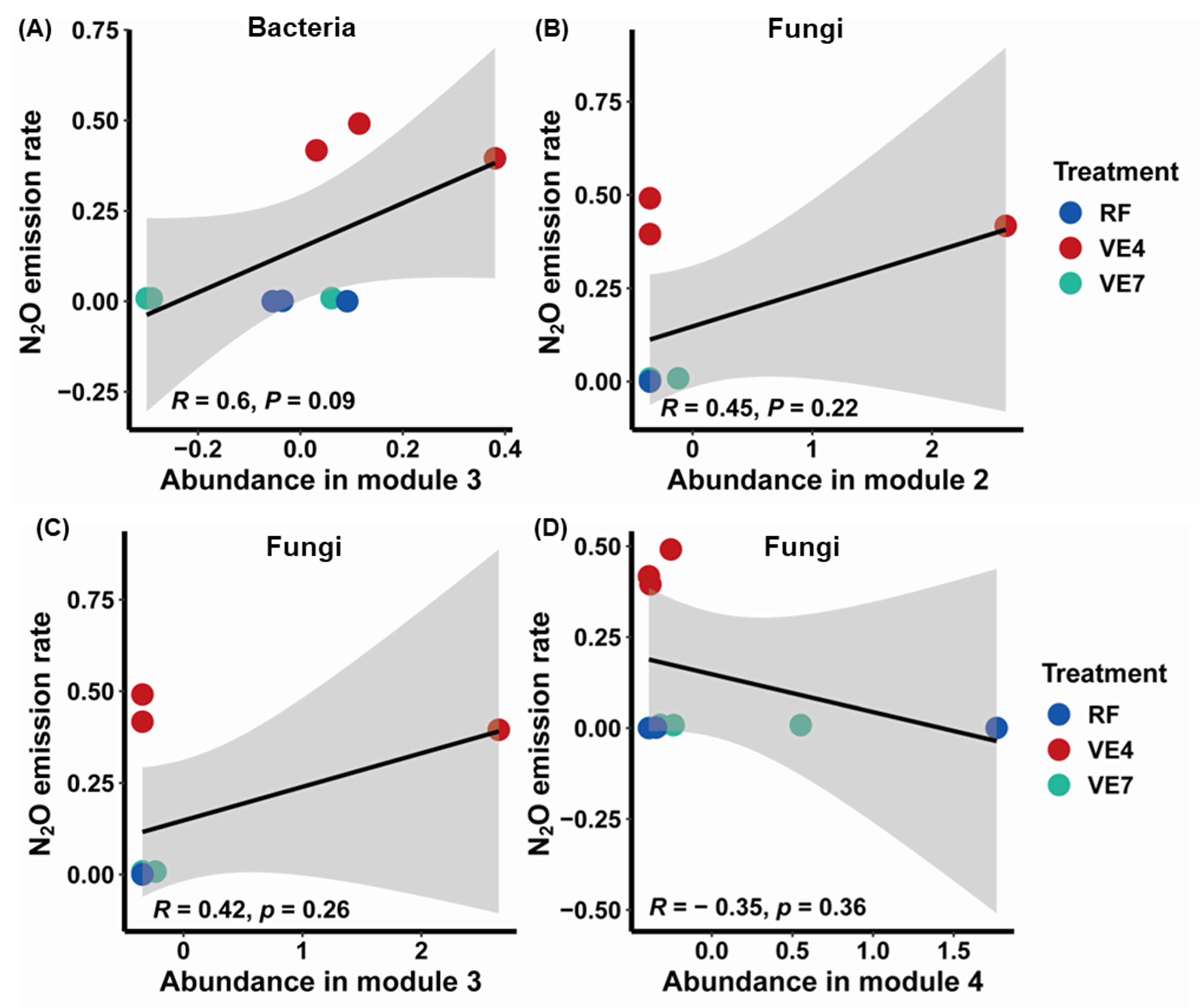
Figure S10. Regression relationships between soil N_2_O emission rates and the relative abundances of bacterial module 2 (A) and fungal modules 2 (B), 3 (C), and 4 (D). Circles in different colors represent the rice field (RF) and vegetable fields converted from the rice field for 4 years (VE4) and 7 years (VE7).


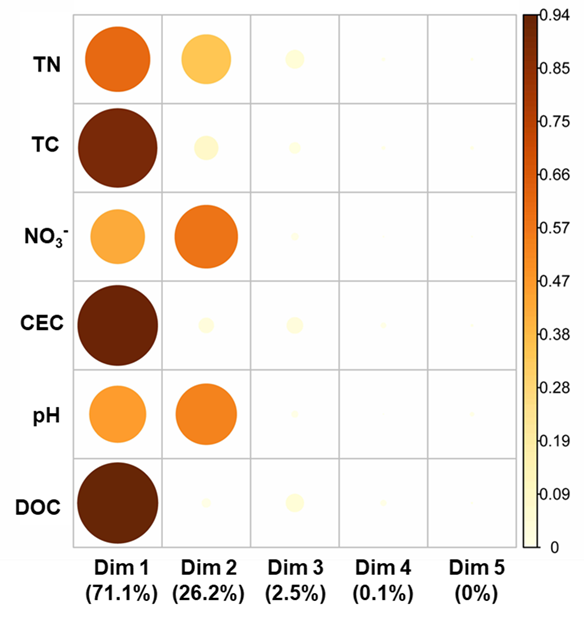


Figure S11. The explanatory ability of different principal components for soil properties obtained from PCA analysis. The red gradient represents the degree of explanatory ability, where darker colors denote higher explanatory strength.


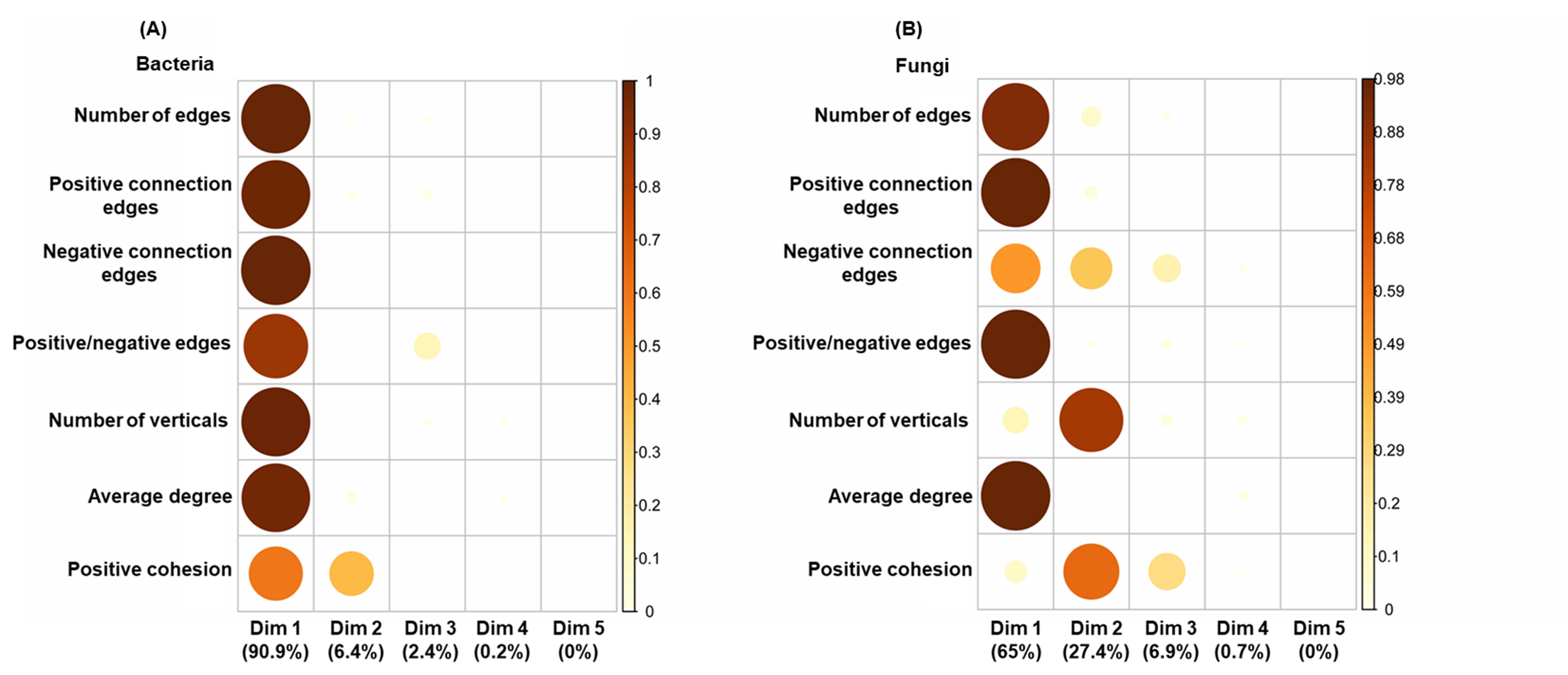


Figure S12. The explanatory ability of different principal components for bacterial (A) and fungal (B) network topological properties obtained from PCA analysis. The red gradient represents the degree of explanatory ability, where darker colors denote higher explanatory strength.


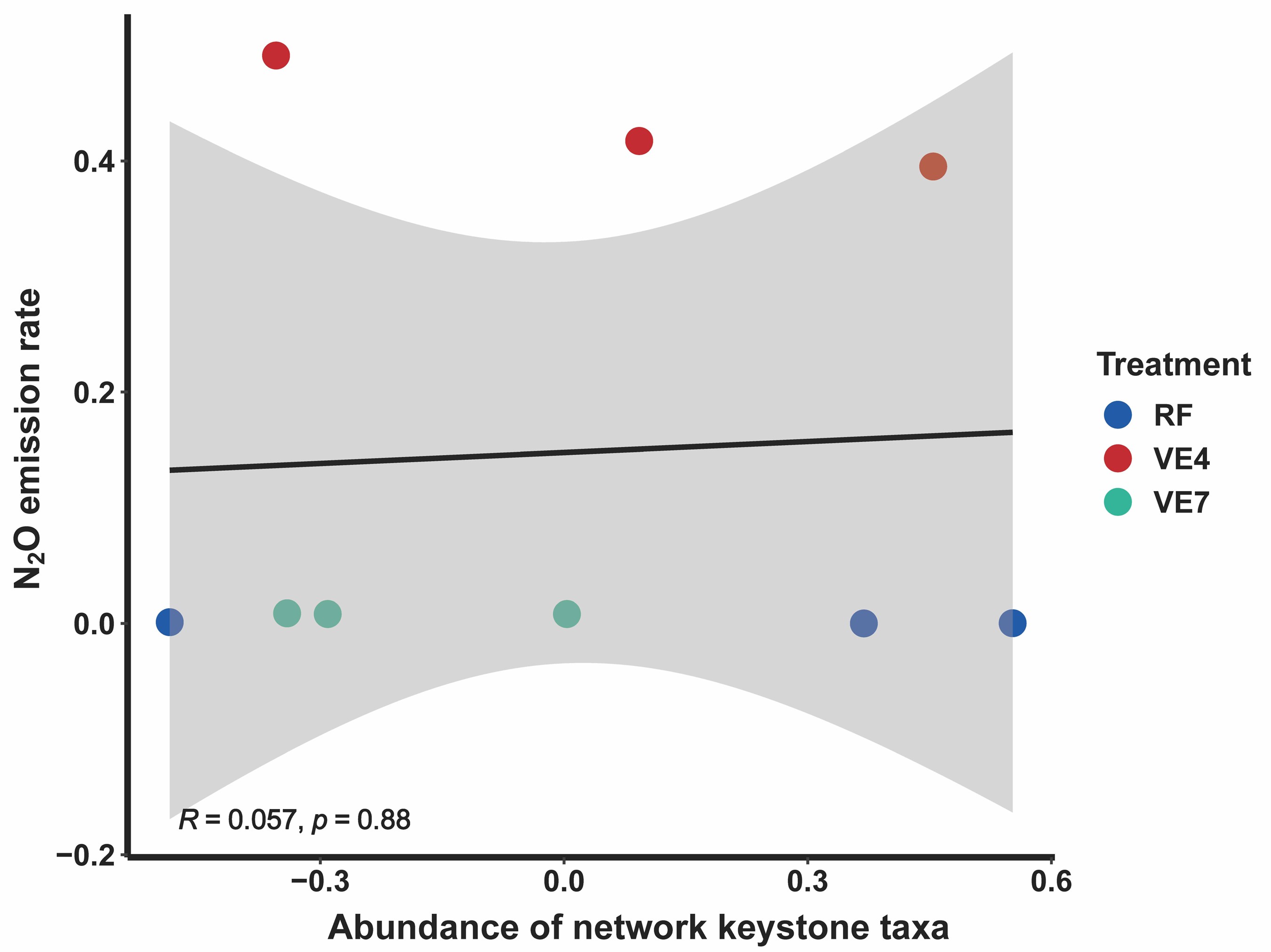
Figure S13. Regression relationships between soil N_2_O emission rates and the relative abundances of network keystone taxa. Circles in different colors represent the rice field (RF) and vegetable fields converted from the rice field for 4 years (VE4) and 7 years (VE7).


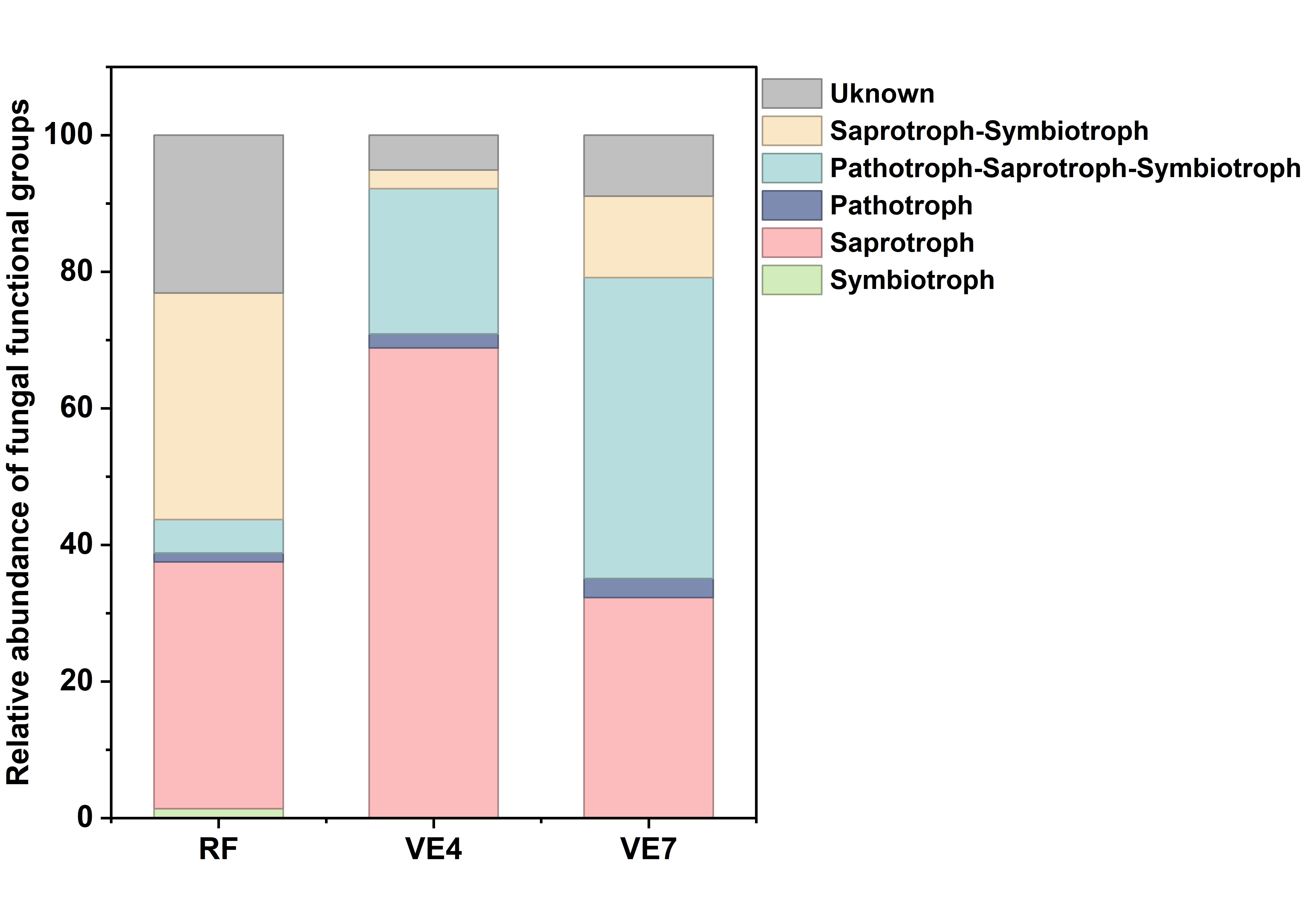


Figure S14. Variations in composition of fungal functional groups inferred by FUNGuild across a rice field (RF) and vegetable fields converted from the rice field for 4 years (VE4) and 7 years (VE7).


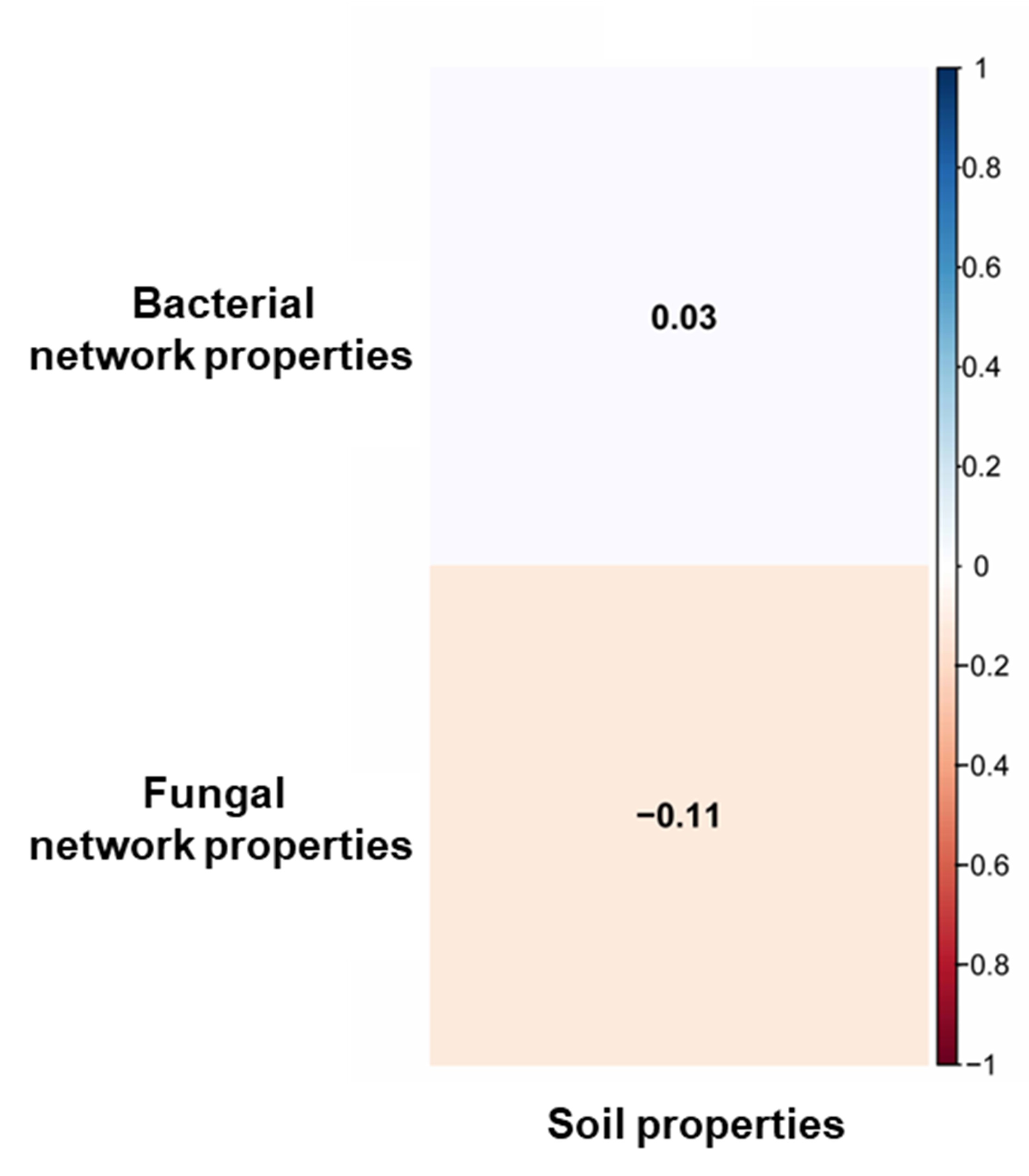


Figure S15. Pearson correlations between soil properties and microbial network characteristics. The color gradient and values denote Pearson’s correlation coefficients.

References

Caporaso, J.G. et al., 2010. QIIME allows analysis of high-throughput community sequencing data. Nat. Methods, 7: 335-336. <https://doi.org/10.1038/nmeth.f.303>

Dong, L.Z. et al., 2024. Microbial diversity is especially important for supporting soil function in low nitrogen ecosystems. Soil Biol. Biochem., 194: 109442. https://doi.org/10.1016/j.soilbio.2024.109442

Wei, Z.J. et al., 2022. Land use conversion and soil moisture affect the magnitude and pattern of soil-borne N_2_, NO, and N_2_O emissions. Geoderma, 407: 115568. <https://doi.org/https://doi.org/10.1016/j.geoderma.2021.115568>

Yu, W.J. et al., 2025. Microbial taxa and interactions can predict lignin mineralization in soil at continental scale. Soil Biol. Biochem., 204: 109763. https://doi.org/10.1016/j.soilbio.2025.109763

Zhu, M.H. et al., 2024. High functional breadth of microbial communities decreases home-field advantage of litter decomposition. Soil Biol. Biochem., 188: 109232. <https://doi.org/10.1016/j.soilbio.2023.109232>
